# Decomposing decision-making in preschoolers: Making decisions under ambiguity versus risk

**DOI:** 10.1371/journal.pone.0311295

**Published:** 2024-09-30

**Authors:** Nancy Garon, Ellen Doucet, Bronwyn Inness

**Affiliations:** Department of Psychology, Mount Allison University, Sackville, NB, Canada; Texas A&M University, UNITED STATES OF AMERICA

## Abstract

Decision-making in the real world involves multiple abilities. The main goal of the current study was to examine the abilities underlying the Preschool Gambling task (PGT), a preschool variant of the Iowa Gambling task (IGT), in the context of an integrative decision-making framework. Preschoolers (*n* = 144) were given the PGT along with four novel decision-making tasks assessing either decision-making under ambiguity or decision-making under risk. Results indicated that the ability to learn from feedback, to maintain a stable preference, and to integrate losses and gains contributed to the variance in decision-making on the PGT. Furthermore, children’s awareness level on the PGT contributed additional variance, suggesting both implicit and explicit processes are involved. The results partially support the integrative decision-making framework and suggest that multiple abilities contribute to individual differences in decision-making on the PGT.

## Introduction

In the 1960’s, Walter Mischel administered a simple decision-making task, the delay of gratification (DoG) task, to preschoolers [1]. While this line of research resulted in a wealth of insight into the mechanisms underlying self-control and decision-making, two insights were particularly important. First, performance on this task was predictive of social and cognitive outcomes in adolescence [2] and in adulthood [3]. Second, strategies children used during the delay period were important in determining their performance [4]. Part of the reason this simple task has been so effective in the prediction of a variety of outcomes is that it taxes an individual’s ability to regulate motivational desires, which is at the heart of many human dilemmas.

However, the DoG task only considers decisions where the outcomes for both options are known. Many life choices involve making decisions when the outcomes are uncertain or unknown. In the case of decisions in ambiguous situations (e.g., a new social situation), the decision maker must first learn through the process of making choices and receiving feedback. Arguably, this type of ambiguous situation is the most difficult decision-making situation and the type of task that has the most potential for providing a valid assessment of how an individual interacts with their environment. Furthermore, it has the potential to contribute to our understanding of developmental disorders that involve social difficulties such as Attention Deficit Hyperactivity Disorder or Autism Spectrum Disorder, disorders that have consistently been associated with decision-making impairments [5–8]. Unfortunately, after a promising beginning twenty years ago, there has been relatively little work done in the past decade on early decision-making in ambiguous situations. As a result, the main goal of the current study was to investigate an integrative decision-making framework using a battery of novel preschool decision-making tasks. This framework [9] posits that different processes are involved in making decisions under ambiguity, when option outcomes are not known, and making decisions under risk, when outcome options are known.

### Decisions under ambiguity: Iowa Gambling task

Bechara et al. [10] created the Iowa Gambling task (IGT) to assess individuals who had lesions in the ventromedial prefrontal cortex (VMPFC). These individuals, despite showing severe problems in their lives, were performing normally on traditional tests of prefrontal lobe functioning. Two main aspects of the IGT capture the difficulty of making decisions in a real situation. First, the task provides no information on the contingencies of the four options and second, the four options present a conflict between short-term and long-term outcomes. This setup leads to two main stages of decision-making. In the first stage, participants must learn the contingencies through feedback (i.e., losses and wins). Second, once participants have learned the contingencies, they can make their choice with knowledge of the conflict in short- and long-term contingencies. Brand et al. [9] called these two stages decision-making under ambiguity and decision-making under risk.

These stages parallel different levels of awareness in the IGT. Bechara et al. [11] found that participants went through three stages as they played the game. In the pre-hunch stage, participants reported not knowing which of the four decks were best but showed increased skin conductance responses before choosing from the disadvantageous decks, suggesting implicit knowledge. In the hunch stage, participants reported that some decks were better than others and showed a significant increase in choice of the advantageous decks. Finally, in the conceptual stage, participants reported knowledge of contingencies, but showed little change in choice behavior. Interestingly, the participants with VMPFC lesions not only showed significantly lower levels of advantageous choice, but also never reached the hunch stage [11]. Even those participants with VMPFC lesions who gained conceptual knowledge of the game, did not change their pattern of choice.

To explain the decision-making difficulties of the participants with VMPFC lesion, Damasio [12] proposed the somatic marker hypothesis whereby the VMPFC acts as a convergence zone in the brain, connecting a category of events to certain somatic states based on experience. More importantly, somatic markers constrain the decision space by reducing a large amount of information into a biasing feeling to make decisions easier.

These stages of decision-making and awareness also parallel exploration and exploitation strategies used in the IGT. Exploration refers to shifting between the choice options; this strategy has been associated with the uncertainty of the situation [13]. Exploitation, on the other hand, refers to a maximizing strategy where participants choose primarily from the option(s) that generates the largest reward in the long-term [14]. Ligneul [15] found that participants tended to show a pattern of exploration in the beginning of the IGT when they were uncertain about the contingencies before switching to an exploitation strategy once they had knowledge of the game.

### Preschool Gambling task

Twenty years ago, Kerr and Zelazo [16] created a preschool version of the IGT, the Children’s Gambling task (CGT), which simplified the original task by reducing the number of decks from four to two and used candy to indicate wins and losses. However, the critical components of the task remained: children were not given information about the contingencies and the two options created a conflict between immediate and delayed outcomes. Kerr and Zelazo [16] found an age difference whereby 4-year-olds chose significantly more from the advantageous deck and 3-year-olds showed a tendency to choose more from the disadvantageous deck. Later studies using this task and other preschool variants also found age differences during the preschool period [17–19].

Research in preschoolers suggests that, like adults, they also demonstrate stages of decision-making. For instance, preschoolers progress through a hunch phase where they identify one deck as better than another, followed by a conceptual phase where they can verbalize that decks differ in terms of losses and gains [17–20]. Furthermore, they show similar strategy and behavior changes during these phases. For instance, they show exploratory behavior during the pre-hunch phase whereby they shift their choice from one deck to the other [21]. Approximately mid-way through the game, once they have accumulated more information, they reduce exploration and increase exploitation [21]. The change in awareness is also accompanied by an increase in choices from the advantageous deck [17–20]. Moreover, the preschoolers who showed this strategy pattern were rated as higher in prosocial behavior by parents [21], suggesting the type of strategy used during the game may provide insight into children’s social worlds.

Research on decision-making in children has been largely done on middle-class populations. While this research has indicated an association between awareness and performance on decision-making tasks, recent research comparing young children from lower and middle social economic status has shown differences in these association indicating that explicit knowledge and task performance may not be associated in child population from lower SES backgrounds [22].

### Current study

The purpose of the current study was to explore the association of new preschool decision-making tasks using the stages of decision-making as a framework. We adapted four tasks that have been previously used in the literature on preschoolers or older children to measure different components of decision-making. We used Multilevel Modelling (MLM) to explore whether these other decision-making tasks were associated with the Preschool Gambling task [PGT; 23], an adapted version of the IGT.

Based on past findings [21], we expected that children would show a shift in strategy during the PGT; this shift would be marked by moderate exploration combined with low exploitation early on and low exploration combined with moderate exploitation later on in the game. We also expected differences in these strategies according to the level of awareness reached, moderate exploration being characteristic of the pre-hunch stage and moderate exploitation being characteristic of the hunch and conceptual stage. Finally, we expected that decision-making tasks involving ambiguity would be associated with the first stage of decision-making while the tasks where contingencies were known would be associated with the second stage of decision-making on the PGT.

#### Stage 1: Decisions under ambiguity

To assess decisions under ambiguity, a passive avoidance task was used for the current study. Passive avoidance tasks have been used in the literature to explore how individuals cope with reward and loss feedback and research indicates that these tasks activate a VMPFC network [24, 25]. The basic task involves the presentation of a stimulus and asking individuals to play or pass on that trial [26]. While participants receive a reward for some stimulus, they experience a loss with others. More importantly for this study, participants are not given information on the contingencies. Difficulties in this task have been associated with antisocial behavior in the adult literature [e.g., 26, 27] and behavioral disorders in children and adolescents [e.g., 24, 25]. Recently, Briggs-Gowan et al. [28] created a computerized preschool version of the task, Stars in Jars, with four types of Jars, two of which resulted in a win and two of which resulted in a loss. Performance on this task was associated with punishment insensitivity. We expected that stronger performance on the passive avoidance task would be associated with stronger performance on the first stage of the PGT. Since optimal performance on this task relied on learning the contingencies, we also expected that stronger performance should also lead to higher awareness level earlier on in the PGT.

#### Stage 2: Decisions under risk

The literature contains more examples of tasks that specifically assess decision-making when the value for each option is known. Perhaps the simplest decision that can be made is choosing which of two options is preferred. Interestingly, these preference consistency tasks have been found to activate a VMPFC network [29, 30]. Presumably, to make consistent choices on this task, the decision maker must have strong, distinct preferences. In support of this idea, Grueschow et al. [31] found that a stronger value coding in the brain was associated with an increase in choice consistency. Furthermore, Lee et al. [32] found that individuals with strong emotions make choices that are more consistent. Typically, consistency in choice is assessed using errors of transitivity [33]. A transitivity error occurs, for example, if an individual with previous choices of A>B and B>C chooses C>A on a subsequent trial. While there is some work on children [34] and adults using this task [32], there is very little work done on preschool-aged children [35]. Each choice in the current transitivity task involved six toys that were paired, with all possible pairings resulting in 15 choices.

Risky decision-making tasks are another major type of task used in the literature to assess choice with knowledge of option values. In the adult literature, some of these tasks have been closely modelled after the original IGT with the exception that probabilities and magnitudes of gains/losses are displayed for each trial. In this type of task, participants typically choose between a safe option where the outcome is certain (e.g., 100% chance of winning $5) and an option in which the outcome is uncertain (e.g., 50% chance of winning $10 and 50% chance of winning $0). The choices are typically framed as a gain or a loss. Performance on this task is usually assessed using the number of risky choices that are advantageous (e.g., result in a higher expected value) versus risky choices that are disadvantageous. A variation of this task, called the Cups Task [36], indicated that while risk-taking in the loss domain remained constant, risk-taking in the gain domain decreased from preschool to adulthood [37]. The current task was inspired by the Cups Task [36].

Finally, a variant of the delay of gratification (DoG) task was used. There have been primarily two types of DoG tasks used in the preschool literature [38]. The first type of task assesses the length of time children are able to delay for a larger reward [1, 39–41], and the second type of DoG task assesses the number of choices to delay for a larger reward [42–47]. There have been some inconsistent findings in age differences using the choice DoG tasks [48, 49]. Part of the issue with this task is that some young children do not consider the temporal dimension of the choice but focus on the magnitude of the two options instead. This can lead to seemingly contradictory findings. Researchers who study self-control in the animal literature have noted such a possibility and have cautioned the use of choice tasks to measure self-control [50]. In the current study, a variation of the hybrid delay of gratification task that is used in animal research [51] was employed to increase the sensitivity of the task to measure age differences in self-control.

The study had five objectives. First, we wanted to investigate whether the simpler decision-making tasks would predict the amount of exploitation on the PGT. Second, we wanted to investigate whether these simpler tasks would predict exploration on the PGT. Third, we wanted to investigate the pattern of exploitation versus exploration on the PGT. Fourth, we wanted to investigate whether these simpler tasks would predict awareness on the PGT. Finally, we wanted to investigate the contribution of awareness to exploitation and exploration on the PGT. It was expected that the passive avoidance task would predict variance in exploitation and exploration in the early stages of the PGT whereas the three tasks with known options would predict variance in the later stages of the PGT. Since the passive avoidance task required learning contingencies, it was expected that higher performance on this task would be associated with higher awareness on the PGT. Finally, it was expected that awareness would predict exploitation and exploration on the latter half of the PGT.

## Method

### Participants

One-hundred-and-forty-four participants were recruited from daycare centers in Eastern Canada. The children were predominantly Caucasian and from middle class families. Not all children finished each task (see Table 1 for *n*’s). The sample consisted of 77 females and 67 males and had a mean age of 49.99 months (SD = 9.20). Children’s PGT data was included if they chose at least two cards from each deck (i.e., they experienced a win and loss for each deck). As a result of this, seven children were excluded (3 males and 4 females). These children did not significantly differ from the other children on the other variables, including gender distribution (all *p*’s > .05).

**Table 1:** Descriptive statistics for the sample.

	Preschool Gambling Task Variables												Other Decision Making Tasks					
	2	3	4	5	6	7	8	9	10	11	12	13	14	15	16	17	18	19
1. Age	.05	.20*	.16	.30**	.08	-.03	-.04	-.05	.16	.22**	.36**	.38**	-.01	.46**	.45**	.33**	.06	.06
2. Exploit1	-	.47**	.26**	.19*	-.48**	-.43**	-.28**	-.14	.07	-.03	.04	.05	-.01	-.05	-.01	.04	.04	-.05
3. Exploit2	.47**	-	.70**	.39**	-.32**	-.67**	-.51**	-.42**	.25**	.13	.30**	.26**	-.06	.17*	.13	.15	.07	.05
4. Exploit3	.26**	.69**	-	.59**	-.21*	-.51**	-.69**	-.56**	.37**	.19*	.35**	.27**	-.04	.24**	.17*	.16	.11	.03
5. Exploit4	.19*	.35**	.58**	-	-.13	-.40**	-.49**	-.62**	.53**	.38**	.38**	.36**	-.02	.22*	.15	.30**	.23**	.01
6. Explore1	-.49**	-.35**	-.23**	-.17	-	.63**	.50**	.36**	-.01	.13	.18*	.11	.09	.14	.13	.09	-.07	-.03
7. Explore2	-.43**	-.68**	-.51**	-.41**	.63**	-	.75**	.62**	-.20*	-.11	-.07	-.09	.18*	-.04	-.01	.02	-.25**	-.12
8. Explore3	-.28**	-.52**	-.70**	-.50**	.51**	.75**	-	.78**	-.27**	-.08	-.15	-.14	.17*	-.16	-.05	-.06	-.15	-.07
9. Explore4	-.14	-.42**	-.56**	-.63**	.36**	.61**	.78**	-	-.23**	-.03	-.16	-.14	.20*	-.17*	-.08	-.12	-.20*	-.01
10.Know1	.07	.22*	.35**	.51**	-.01	-.20*	-.26**	-.23**	-	.43**	.53**	.39**	-.06	.19*	.16*	.27**	.02	-.07
11.Know2	-.04	.09	.16	.34**	.12	-10	-.07	-.02	.41**	-	.35**	.50**	.14	.19*	.12	.19**	.11	-.06
12.Why1	.03	.25**	.31**	.30**	.16	-.06	-.15	-.15	.51**	.30**	-	.83**	-.02	.26**	.30**	.35**	.10	-.07
13.Why2	.03	.21*	.23**	.28**	.08	-.08	-.14	-.13	.36**	.46**	.80**	-	-.03	.24**	.27**	.27**	.11	-.02
14. Trans err	-.01	-.06	-.04	-.02	.09	.18*	.17*	.20*	-.06	.14	-.01	-.02	-	-.07	-.19*	.04	-.04	-.04
15. PA1	-.08	.09	.19*	.10	.12	-.03	-.16	-.17	.13	.10	.11	.08	-.07	-	.58**	.35**	-.01	-.01
16. PA2	-.03	.05	.11	.02	.11	.01	-.03	-.06	.10	.03	.16	.12	-.20**	.46**	-	.21*	.08	-.08
17. DoG	.03	.09	.11	.23**	.07	.03	-.05	-.11	.23*	.13	.26**	.17*	.04	.24**	.08	-	.02	-.13
18. RDloss	.04	.06	.10	.22**	-.07	-.25**	-.15	-.19*	.02	.10	.08	.10	-.04	-.04	.06	-.01	-	.17*
19. RDgain	-.05	.04	.02	-.01	-.03	-.12	-.07	.01	-.08	-.08	-.10	-.04	-.04	-.05	-.12	-.16	.17	-
n	134	134	134	134	134	134	134	134	134	134	134	134	143	144	144	140	140	141
Mean	.25	.44	.51	.57	.47	.34	.29	.22	1.35	1.41	.67	.67	.20	.63	.65	.51	.43	.50
S.D.	.26	.37	.42	.44	.29	.31	.33	.31	.90	.85	.90	.92	.13	.16	.19	.38	.17	.22

Abbreviations: Exploit = Exploitation, Explore = Exploration, PA = Passive Avoidance D-prime score, DoG = Delay of Gratification, RDloss = Risky Decision Making, loss frame, RDgain = Risky Decision Making, gain frame. Age in months partial correlations appear below the diagonal. *p < .05

**p < .01.

### Materials

For the PGT, two decks of 40 cards were used. While previous research on preschool-adapted versions of the IGT have traditionally used 50 trials, it was decided for this study to use 40 trials. This was due to two considerations. First, previous research by the authors on 124 preschool participants indicated that Block 3 and 4 on the PGT explained approximately 86.3% of the variance in the final score of a 5-block PGT and that adding a fifth block did not significantly predict additional variance (*F* (1, 119) = .213, *p* = .645). Second, the battery consisted of five tasks with several trials and it was a concern that some of the younger children would find 50 trials demanding.

**Fig 1:**
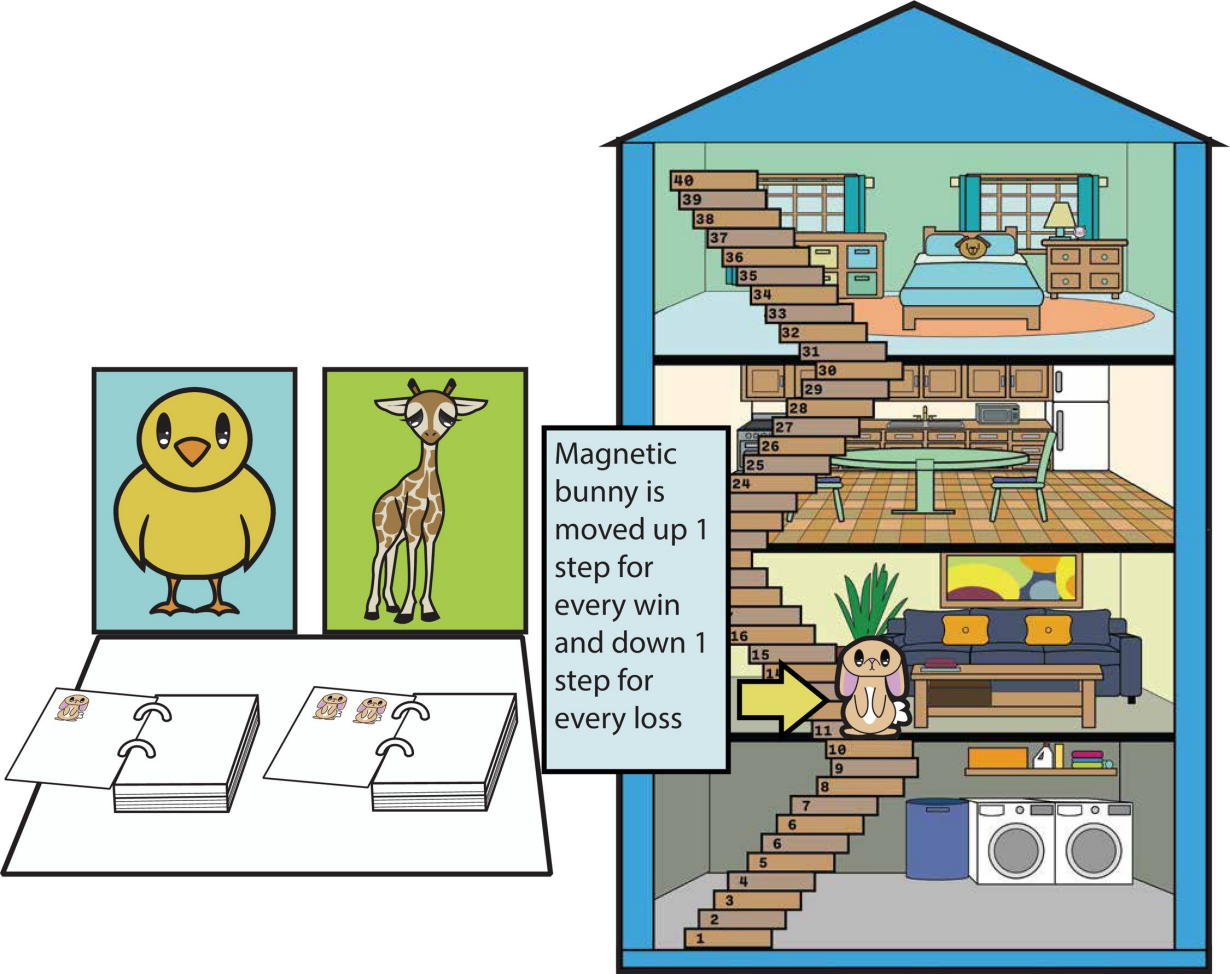
Set up for Preschool Gambling task. Children chose from either the chick or giraffe decks (left). Losses and gains were marked by moving the magnetic marker up and down the stairs (right). Note that the illustration of materials is similar but not identical to the original materials used and is for illustrative purposes.

The win/loss schedule was identical to that used by [23] for the advantageous and disadvantageous decks. Each deck was attached to a felt animal. The advantageous deck was attached to a chick and the disadvantageous deck to a giraffe. Fig 1 shows the set up for the game. A variety of stickers were used for rewards and a magnetic house with stairs (40 stairs in all) was used along with a magnetic marker to show the current number of stickers the child had won. Cronbach alpha for the 40 trials showed good internal reliability, α = .889.

**Fig 2:**
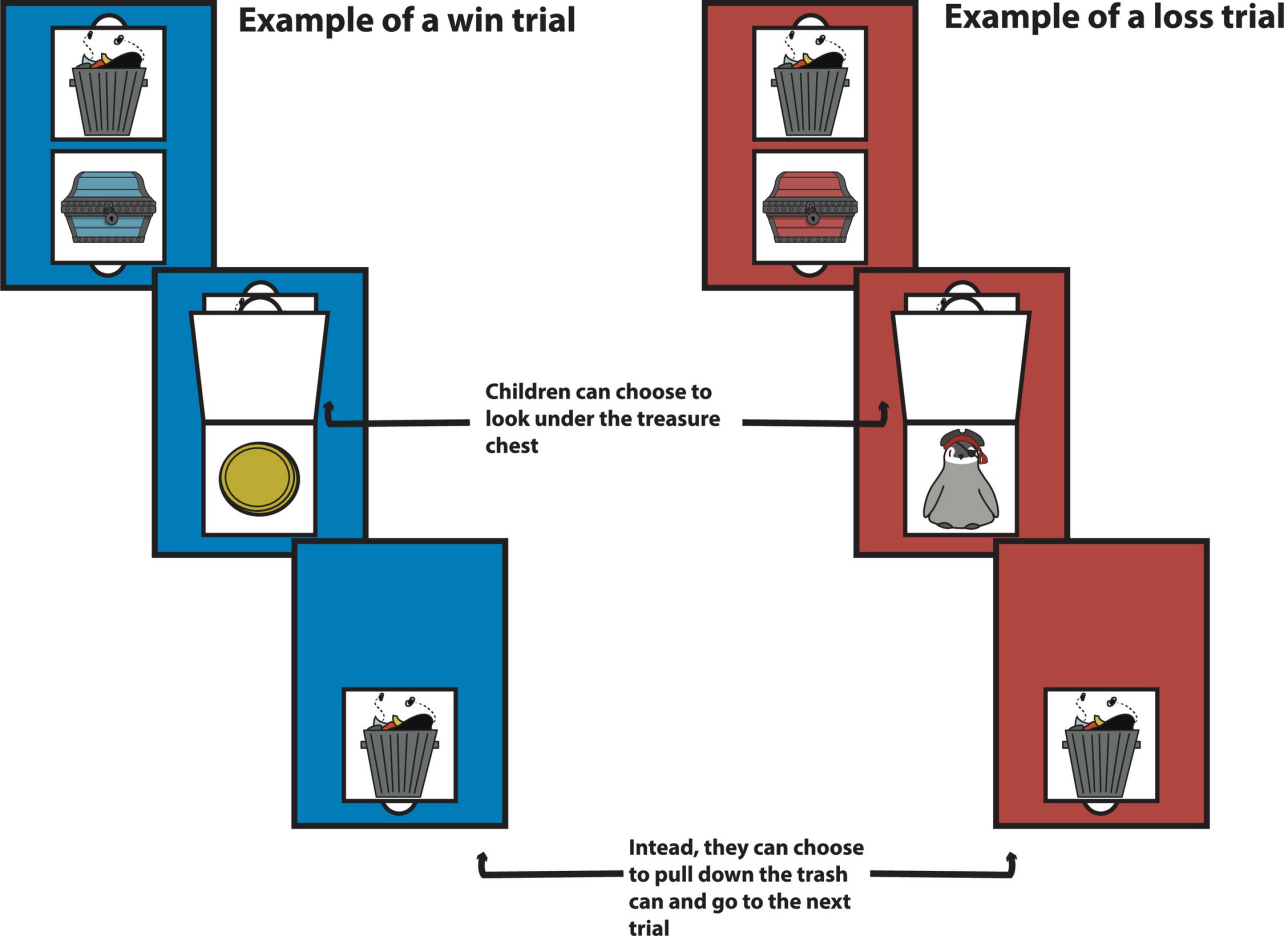
Set up for the Trash to Treasure task (passive avoidance). Children could either pull up the treasure chest to see if there was a gold coin or pull down the trash can to move on to the next trial. Note that the illustration of materials is similar but not identical to the original materials used and is for illustrative purposes.

The passive avoidance task consisted of a flipbook with 40 pages, one demonstration sheet, gold coins, and two containers for the gold coins. There were four different page colors (green, red, yellow and blue) in the flipbook, with each color associated with a different treasure box. Each page had a trash can and a treasure box. The trash can was at the top and could be pulled down over the treasure box if the child decided to pass, while the treasure box could be flipped up. Depending on the type of trial, a gold coin or pirate appeared beneath (see Fig 2). Cronbach alpha for the 40 trials showed very good internal reliability, α = .938.

**Fig 3:**
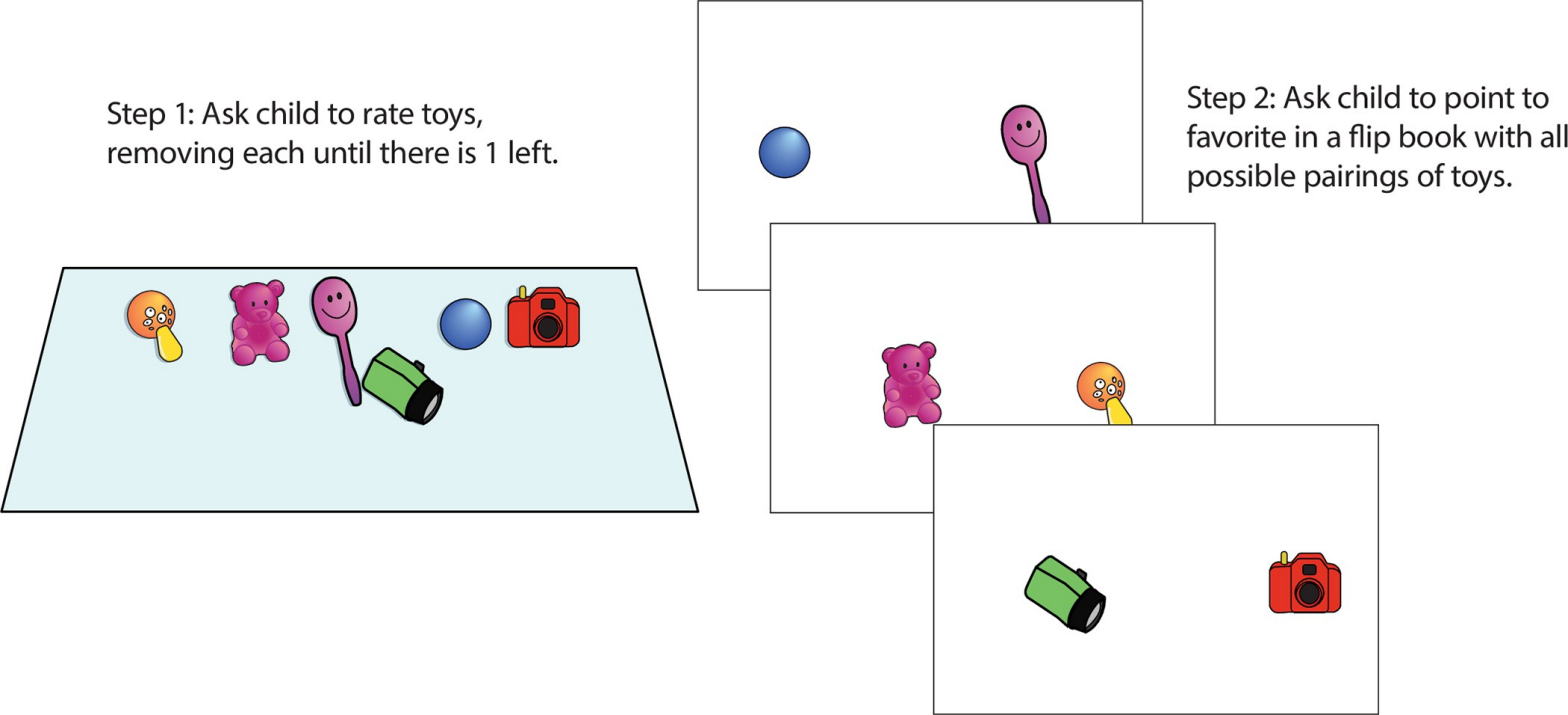
Set up for the Toy Choice task (choice consistency). Following the rating of 6 toys, children pointed to their favorite of two toys. Note that the illustration of materials is similar but not identical to the original materials used and is for illustrative purposes.

The Toy Choice task (preference consistency) involved six toys for rating and a flip book with 15 trials (see Fig 3). Cronbach alpha for the 15 trials showed good internal reliability, α = .846.

**Fig 4:**
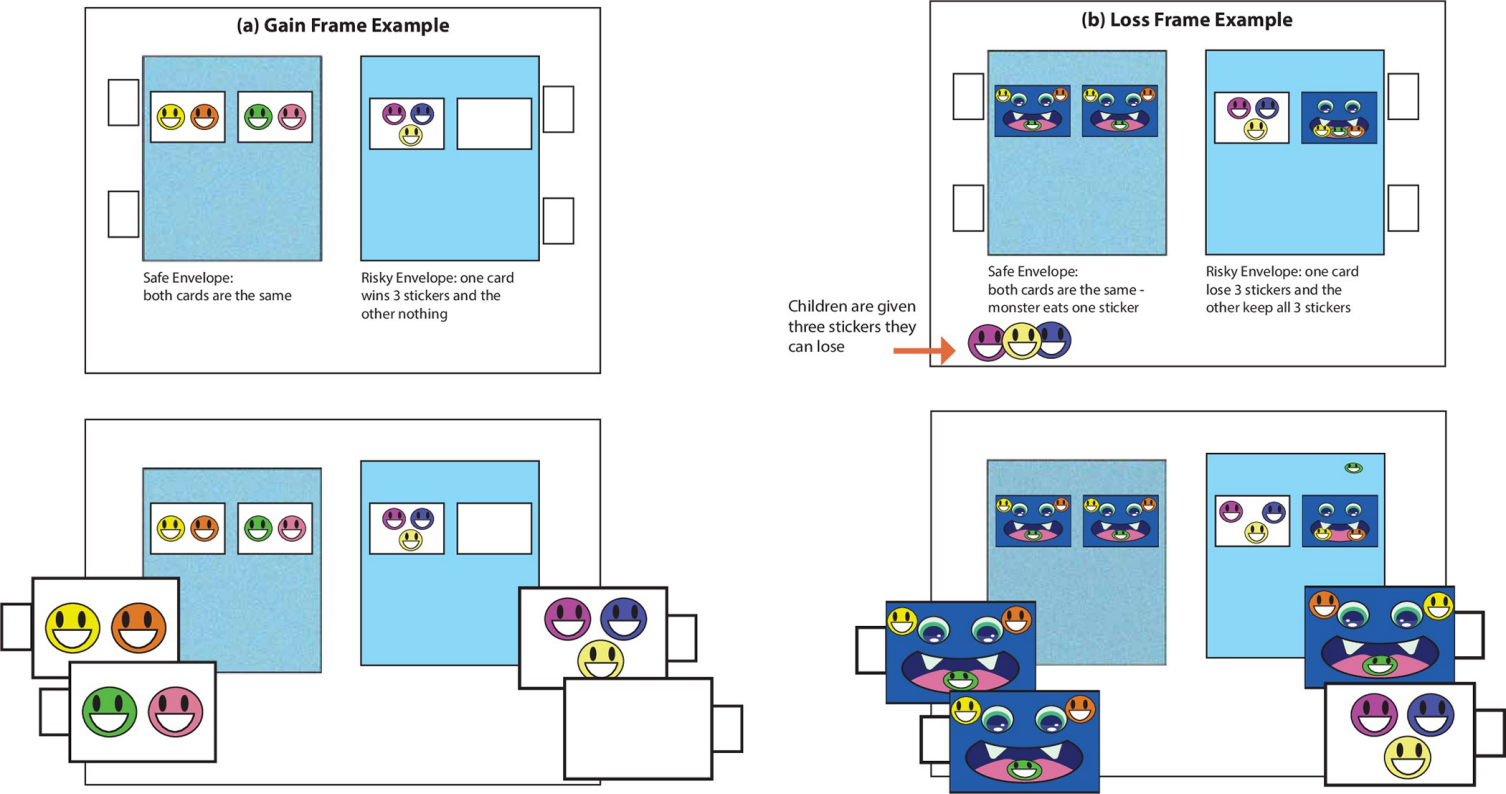
Set up for the Risky Sticker task (risky decision-making). Example (a) shows a trial using the gain frame while example (b) shows a trial using the loss frame. Children were asked to choose from either the safe or risky envelope. While the two possible options were illustrated on the envelope, children did not know which option they were getting until they chose one of the cards. Note that the illustration of materials is similar but not identical to the original materials used and is for illustrative purposes.

The risky decision-making task consisted of a flipbook with 20 pages. Each page had a safe and risky envelope (side was counterbalanced across trials) with two slots for cards. Illustration of the wins/losses appeared on each envelope, but the actual cards for each envelope were hidden in the slots (see Fig 4). Cronbach alpha for the 18 trials showed good internal reliability, α = .814.

**Fig 5:**
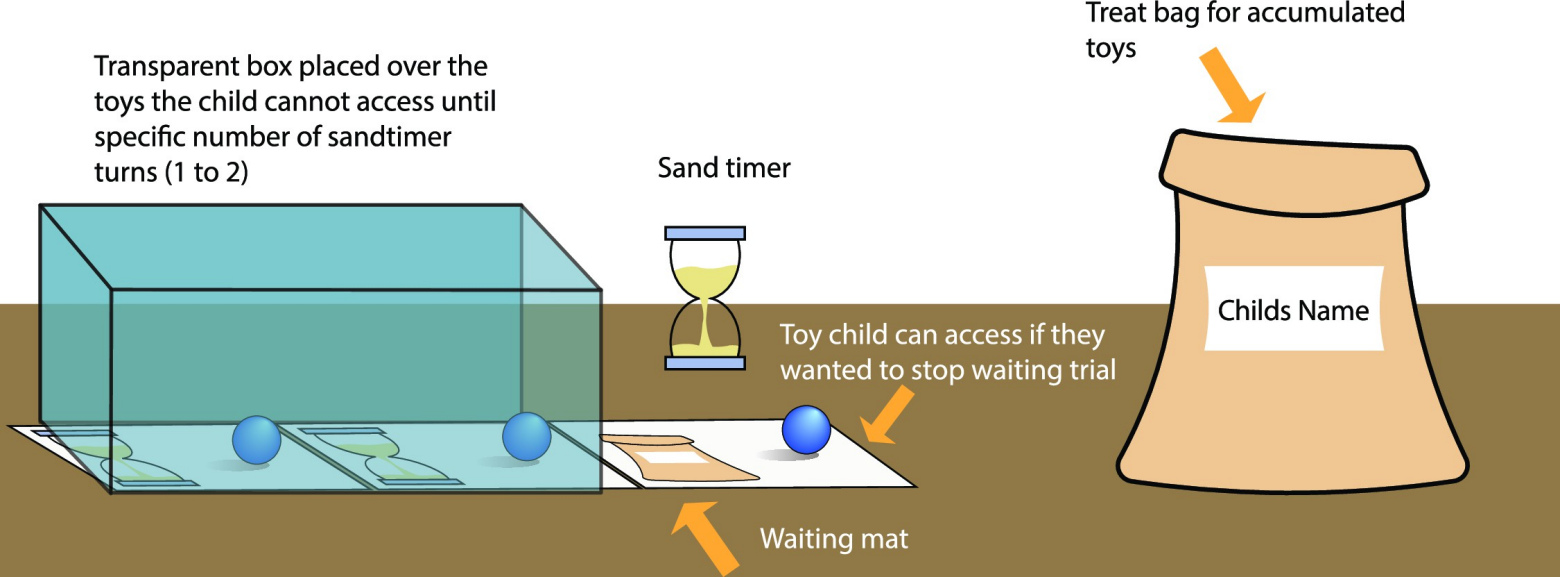
Waiting Game task (delay of gratification). Children were given a paper bag for their accumulated toys. The transparent box was placed over the toys, which required a waiting period to earn. Note that the illustration of materials is similar but not identical to the original materials used and is for illustrative purposes.

The delay of gratification task (DoG) consisted of a transparent box that was placed on a mat displaying different waiting times and a paper bag (see Fig 5). A paper bag was used for their accumulated toys. A puppet was used to demonstrate the task to children. A sand timer to mark waiting times and a variety of small toys were used for this task. Cronbach alpha for the 6 trials showed very good internal reliability, α = .920.

### Procedure

The study was approved by the Mount Allison Research Ethics Board on October 12, 2017. Parents provided written consent for the study. Prior to participation, children were given a child-friendly summary of the tasks and provided verbal assent before each session. Children were tested in a quiet room of their daycare by two experimenters. The decision-making battery took approximately 1 hour to administer and was divided into two sessions of 30 minutes each. Data collection began on November 6, 2017 and ended on July 16, 2018.

#### Preschool Gambling task

Children were told that they would be playing a game where they were going to try to earn as many stickers as possible. They were told that they would choose cards from the chick and giraffe to play this game and were shown the magnetic house and a demonstration card. They were told they would go up one step for each bunny face and go down a step for each monster they saw on the cards. They were told that they would start in the living room and would try to go up as high as they could because the experimenter would give them one sticker for each step they climbed. For each trial, the experimenter called out the wins followed by the losses. Subsequently, they would move the marker up the number of steps corresponding to the wins (1–2 depending on the deck) and go down the number of steps corresponding to losses (0–6 depending on the deck and trial). At the end of the game, children were given the stickers they had won.

Following thirty card choices, the child was given an awareness test consisting of four questions. The first two pertained to the “hunch phase” as children were asked to point to which animal was the best to pick from, and to which animal was the worst to pick from. The next two questions pertained to the “conceptual phase” as children were asked why they thought the animal was the best (the one they identified) and why they thought the animal was the worst. The awareness test was given again following the final 10 card choices.

#### Trash to Treasure task: Passive avoidance

Children were first shown a demonstration card, which had an example of each trial type. They were told that they were going to choose whether to look under a treasure box or put the treasure box in the trash. In the first demonstration trial, the researcher lifted the treasure flap to reveal a gold coin. Children were told that each time they saw a gold coin under a treasure chest, they would get a gold coin and that if they got enough gold coins, they would get a prize. The researcher then went to the next demonstration trial and told the child they could choose to put the treasure chest in the trash if they did not want to play that trial. After demonstrating pulling the trash can down, the researcher lifted the treasure chest to reveal a pirate. The researcher then told the child that some types of treasure boxes had pirates underneath rather than coins and that if this happened, they would lose one of their gold coins. Before proceeding to the task, the researcher checked children’s understanding by asking what happens when they find a gold coin and what happens when they find a pirate. Finally, the researcher asked what they should do if they did not want to open a treasure chest.

Once the researcher was satisfied that the children understood the task, the trials began. The researcher reminded children that the goal of the game was to get as many coins as possible. For each trial, the researcher asked children what they wanted to do. If the child chose to lift the treasure flap, the researcher noted whether the child won or lost a gold coin. If the child chose to pull down the trash flap, the researcher moved to the next trial. There were 40 trials in total. Choosing from either the blue or yellow treasure chests resulted in a gold coin whereas choosing from the red or green treasure chests resulted in a pirate.

#### Toy Choice task: Preference consistency

There were two stages to this task: a rating stage and a binary choice stage. The researcher first showed children 6 toys and asked them to choose their favorite toy. This toy was put aside, and the researcher asked children to choose their favorite among the remaining toys and so on until all toys had been ranked. For each binary choice after the ranking stage, children were shown two side by side pictures of the toys (side counterbalanced across trials) and asked to point to their favorite. There were 15 trials in all, with all possible binary combinations of the 6 toys presented.

#### Risky Sticker task: Risky decision-making

The gain frame trials were presented first followed by the loss frame trials. The researcher showed children the first demonstration trial and explained that children would be choosing from one of the envelopes. The researcher pointed to an illustration of possible cards on each envelope and pulled out the two cards in the risky envelope to demonstrate that the illustrations showed the cards in the corresponding envelopes (see Fig 4). The researcher then described what would happen with each card (i.e., get three stickers with this one and get no stickers with this one). The researcher then did the same thing with the safe side, pointing out that the two cards were the same. After replacing the cards for each envelope, the researcher asked the child to pick a card. Once the child chose a card, the researcher called out how many stickers the child had won and gave them the number of stickers. The remaining trials proceeded in this way with the researcher calling out the outcome and giving children the number of stickers that they won. For the loss frame trials, the children were asked to choose a card from either the safe or the risky side. Like the gain frame, the researcher demonstrated a trial while explaining outcomes for each card. Unlike the gain frame trials, in the loss frame trials, children were first given a set number of stickers. There were 2 demonstration trials and 18 test trials.

#### Waiting Game task: Delay of gratification

For the demonstration trial, the researcher placed one sticker on each of the three divisions of the waiting mat (see Fig 5). The last two divisions had an illustration of the sand timer to indicate children would have to wait for toys placed on these divisions. A transparent box covered these last two divisions to show children they did not have access to these toys during wait times. The sand timer was placed in front of the child. The researcher told the child they were going to play a deciding game. They told the child that the sand timer was going to help them see the wait times and that Lambchop (puppet) was going to show them how the game is played. For the demonstration trial, the researcher told the puppet that they could have one sticker if they did not want to wait, two stickers if they wanted to wait one sand timer turn, and three stickers if they wanted to wait two sand timer turns. For the first demonstration, the puppet indicated that they wanted to wait. The researcher turned the sand timer and once one turn was up (30 seconds), lifted the transparent box, removed the sticker from the middle division and placed it on the first division next to the other sticker. The researcher then asked the puppet again whether they wanted to wait one more time for an additional sticker. The puppet declined and was given the two stickers. The game was reset and in the next demonstration trial, the puppet stated that they did not want to wait and was given one sticker.

The 6 trials were played in this manner, but with different types of small toys and varying numbers of small toys in each of the three divisions. For example, in Trial 1, children could get one ring right away, two rings for 30 seconds, and three rings for waiting 60 seconds. In contrast, in Trial 6, children could get three erasers right away, four erasers for waiting 30 seconds, and five erasers for a wait of 60 seconds. Children were given a paper bag at the beginning of the game and told that it was their bag where they could put all the toys they accumulated from the game.

### Calculation of study variables

#### Preschool Gambling task variables

Exploitation and exploration scores were calculated for each of four blocks of 10 choices. An instance of explore was defined by two consecutive choices, each from a different deck. An example of this would be a choice from the advantageous (A) deck, followed by a choice from the disadvantageous (D) deck, with two of the four possible combinations qualifying as an instance of explore (AD, DA vs AA, DD). Proportion of exploration for each block was calculated using the formula: (#explore for 10 cards)/(#paired trials for 10 cards). An instance of exploitation was defined as choosing from the advantageous deck on two consecutive trials (i.e, AA). Proportion of exploitation for each block was calculated using the formula: (#exploit for 10 cards)/(#paired trials for 10 cards).

Table 2 shows an example of exploitation calculated for Block 1. Choices were grouped into overlapping trial pairs. The number of trial pairs served as the denominator. Note that for Block 1, there are 9 trial pairs, but for the subsequent blocks, there are 10 trial pairs. Each trial pair was scored a “1” for an instance of exploitation or a “0” for no exploitation (see Table 2). In the example provided, there were only two trial pairs scored as a 1. This led to a total block score of 2 divided by 9 for a Block 1 exploitation score of .22. Similarly, trial pairs were used to calculate exploration. For exploration, paired trials were scored 1 if the second choice differed from the first (e.g. AD or DA). In the example provided, there were 5 instances of exploration. Dividing this by the total number of trial pairs results in a Block 1 exploration score of .56.

**Table 2:** Example of exploitation/exploration calculations.

Block 1: Trial											
Trial	1	2	3	4	5	6	7	8	9	10	
Choices	A	A	D	D	D	A	D	A	A	D	
**Paired Trials Score**											
Trial Pairs		1–2	2–3	3–4	4–5	5–6	6–7	7–8	8–9	9–10	
Exploit		1	0	0	0	0	0	0	1	0	2/9 = .22
Explore		0	1	0	0	1	1	1	0	1	5/9 = .56

Abbreviations: A = Advantageous Deck, D = Disadvantageous Deck. Pair 1–2 is an example of exploitation whereas Pair 7–8 is an example of exploration where there was a switch from the disadvantageous deck to the advantageous deck.

For the awareness test variables, children were given 1 point each for correct identification of the helpful/not helpful animal for a total of 2 points (hunch phase). Children were given 1 point each for correctly identifying why the animal was helpful/not helpful (concept phase). They were awarded points for answers that indicated they knew the best animal had more wins than losses and that the worse animal had more losses than wins for a possible total of 2 points.

#### Trash to Treasure task variables

The dependent measure used was D-prime (D’) derived from signal detection theory [52], which is calculated using hits (opening the treasure chest when there was a coin) and false alarms (opening the treasure chest when there was a pirate). Higher values indicate that children were better at identifying the treasure boxes that had gold coins. D-prime was calculated for the first 20 trials (PA1) and the last 20 trials (PA2). Both variables were positively skewed so box-cox transformation was used to address this [53].

#### Toy Choice task variable

The dependent measure used for this task was the proportion of transitivity errors (Trans Err). First, every combination of triplets of options *(A*, *B*, *C)* for the six toys were considered (for the current study, this was 20 triplets). For each triplet of options, there were a total of eight possible choice patterns, of which two were transitivity errors (i.e., violated transitivity), two were transitive, and four were nontransitive [54]. The proportion of errors was calculated by dividing the total number of transitivity errors by the total number of possible errors.

#### Risky Sticker task variables

An expected value (EV) sensitivity score was calculated separately for the loss (RD Loss) and gain domain (RD Gain) by subtracting the number of disadvantageous risky choices from the advantageous risky choices [55]. Scores could range from -3 to 3. Scores were transformed to a proportion score for ease of interpretation by rescaling the scores to range from 0 to 6 and dividing the subsequent score by 6.

#### Waiting Game task variable

Children were given a score of 0 if they did not wait, a score of 1 if they waited 30 seconds, and a score of 2 if children waited 60 seconds. Scores ranged from 0 to 12. Scores were transformed to a proportion score for ease of interpretation by dividing the subsequent score by 12.

### Data analysis

Preliminary analyses (correlations, descriptive statistics, and assumptions analysis) were conducted using SPSS Version 24. To explore the association of the four new decision-making tasks with the decision- making strategies used in the PGT (i.e., exploit and explore), multilevel modeling was used. Due to the number of decision-making variables serving as potential independent variables, preliminary analyses were run to investigate which would contribute to the variance of exploitation and exploration. For exploitation, only three (first half of passive avoidance (PA1), delay of gratification (DoG), and risky decision-making, loss frame (RD Loss) of the six independent variables contributed significantly to the prediction of exploitation. For exploration, only transitivity errors (Trans Err), PA1, and RD Loss contributed significantly to the variance.

For both multilevel analyses (MLM), the intercept and block slope were entered as random variables whereas other predictors were entered as fixed variables. In other words, each child had their own intercept and slope across blocks. A dummy variable was created for test time to explore the impact of asking children the awareness questions at the end of Block 3 on subsequent performance. Previous findings have indicated that asking the awareness questions has an impact on subsequent choice [17]. The blocks occurring before the test were coded as “0” while the block following the test was coded as “1”. Test time was added as a random variable but retained only if it contributed significantly to the variance.

To investigate the pattern of exploration versus exploitation, the simple slopes for exploitation and exploration derived from the MLM analyses were compared to one another. Of particular interest was the point at which there was a transition from exploration to exploitation (i.e., block when exploitation was significantly higher in comparison to exploration).

For awareness, a similar strategy was used. Preliminary analysis indicated that only DoG contributed significantly to the variance. To explore the association of these tasks with awareness in the PGT, two random variables, aware type (hunch vs. conceptual) and test time (after 30 cards vs end of game) were entered to the intercept-only model. Main effects of age in months (age) and the decision-making variables were added along with the interaction of each variable with aware type and test time.

To examine the contribution of explicit awareness on exploitation and exploration, an awareness variable (aware level) was added to the final multilevel models. To construct aware level, children who could not correctly identify which deck was best and which was worse were given a score of 0, those who could correctly identify decks but did not know why were given a score of 1, while children who could provide some explanation of why one deck was better than the other were given a score of 2. These roughly corresponded to the pre-hunch, hunch and conceptual stages. We were interested in two main issues. First, we wanted to examine whether explicit awareness predicted additional variance beyond the decision-making variables already included in the final model. Second, we wanted to examine whether explicit awareness moderated the effect of the decision-making predictors. We first added aware level and its interaction with block, and for the exploitation analysis, its interaction with test time. Next, we added interaction effects of aware level with the decision-making predictors.

For all multilevel models, we used maximum likelihood for estimation. Multilevel modelling has considerable flexibility in modeling continuous predictors, allows for comparison of slopes, and enables the partition of variance into within subject and between subject variance, enabling the exploration of the amount of variance explained by each predictor [56]. All independent variables were centered by subtracting the mean. All models were calculated using the mixed model function of SPSS Version 24. Since each model was nested within the previous one, they could be compared by testing the deviance statistic [57]. Significant interactions were followed up with simple slopes as recommended by Preacher et al. [58].

## Results

Table 1 presents correlations and descriptive statistics for the variables used in the present study. Note that exploitation on the third block was significantly associated with the first half of the passive avoidance task, while exploitation on the fourth block was significantly associated with performance on DoG and RD Loss. In contrast, exploration in the second, third, and fourth block was associated with a higher number of transitivity errors (Trans Err).

### Prediction of exploitation on the PGT

The addition of block in Model B (Table 3) significantly improved model fit, χ^2^ (3) = 123.450, *p* < .001, explaining 37.4% of within-person variation. The addition of test time (Model C) also improved model fit, χ^2^ (4) = 40.64, *p* < .001, explaining an additional 22.2% of within-person variation in exploitation. Adding the main effects of the decision-making variables and age did not significantly improve model fit (Model D), χ^2^ (4) = 8.943, *p* = .063. However, adding the interactions with block and test time did improve fit (Model E), χ^2^ (12) = 196.654, *p* < .001. Since many of the variables were non-significant, the model was pruned to improve power to detect significance. This simpler model (Model F) did not significantly differ from Model E, χ^2^ (5) = 5.875, *p* = .319. However, Model F significantly improved model fit when compared to Model C, χ^2^ (7) = 190.779, *p* < .001. Model F was therefore retained as the final model. This model accounted for 59.6% of within-person variance, 1.1% of between-person variance in exploitation, 11.9% of the variance in changes across the blocks, and 5.9% of variance in change in exploitation following the awareness test.

**Table 3:** Summary of multilevel modeling for exploitation as the dependent measure.

Effect	Model A	Model B	Model C	Model D	Model E	Model F	Model G	Model H
*Fixed Effects*								
Intercept	.445***	.290***	.272***	.272***	.272***	**.273** ***	**.337** **	.339
Block		.103***	.131***	.131***	.131***	**.130** ***	**-.045**	-.045
Test Time			-.093*	-.093*	-.091*	**-.088ŧ**	**.232**	.232
Age				.005	.003			
PA1				-.025	-.148	**-.099**	**-.104**	-.109
DoG				.094	.040	**.062**	**.022**	.026
RD Loss				.191	.052	**.099**	**.086**	.087
Block x Age					-.001			
Block x PA1					.352*	**.369** **	**.277** *	.277*
Block x DoG					.023			
Block x RD Loss					.104			
Test Time x Age					.008			
Test Time x PA1					-.705*	**-.603** ŧ	**-.510**	-.510
Test Time x DoG					.144	**.213** *	**.148**	.148
Test Time x RD Loss					.216	**.408** *	**.389** *	.389*
Aware Level							**.031**	.020
Block x Aware Level							**.070** **	.070**
Test Time x Aware Level							**-.027**	-.027
Aware Level x PA1								.015
Aware Level x DoG								.003
Aware Level x RD Loss								.107
*Random Effects*								
Within-person	.100***	.063***	.041***	.041***	.041***	**.041** ***	**.041** ***	.041***
Initial status	.057***	.037**	.037***	.038***	.036***	**.036** ***	**.036** ***	.036***
Block effect		.012***	.026***	.026***	.023***	**.023** ***	**.020** ***	.020***
Test effect			.140***	.140***	.128***	**.131** ***	**.131** ***	.131***
*Pseudo R* ^*2*^								
Within-person		.374	.596	.596	.596	**.596**	**.596**	.596
Initial Status				.000	.025	**.011**	**.027**	.033
Block effect				.000	.134	**.119**	**.250**	.250
Test Time effect				.000	.084	**.059**	**.064**	.064
*Deviance*	440.594	317.144	276.502	267.559	243.940	**249.815**	**220.540**	219.904
AIC	446.594	329.144	296.502	295.559	287.940	**283.815**	**260.540**	265.904
BIC	459.401	354.758	339.193	355.326	381.860	**356.389**	**345.922**	364.093
Model test		A vs B	B vs C	C vs D	D vs E	**E vs F**	**F vs G**	G vs H
Significance (χ^2^)		χ^2^ (3) = 123.450***	χ^2^ (4) = 40.642***	χ2 (4) = 8.943	χ^2^ (8) = 23.619**	**χ**^**2**^ **(5) = 5.875**	**χ**^**2**^ (3) = 29.275^***^	χ^2^ (3) = 0.888
					C vs E	**C vs F**		
					χ2 (12) = 196.654***	**χ2 (7) = 190.779** ^***^		

Abbreviations: PA1 = Passive Avoidance first half, D-prime score, DoG = Delay of Gratification, RD Loss = Risky Decision Making, loss frame, Aware = Awareness. Two final models appear in bold.

ŧ *p* < .1

*p < .05

**p < .01

***p < .001.

**Fig 6:**
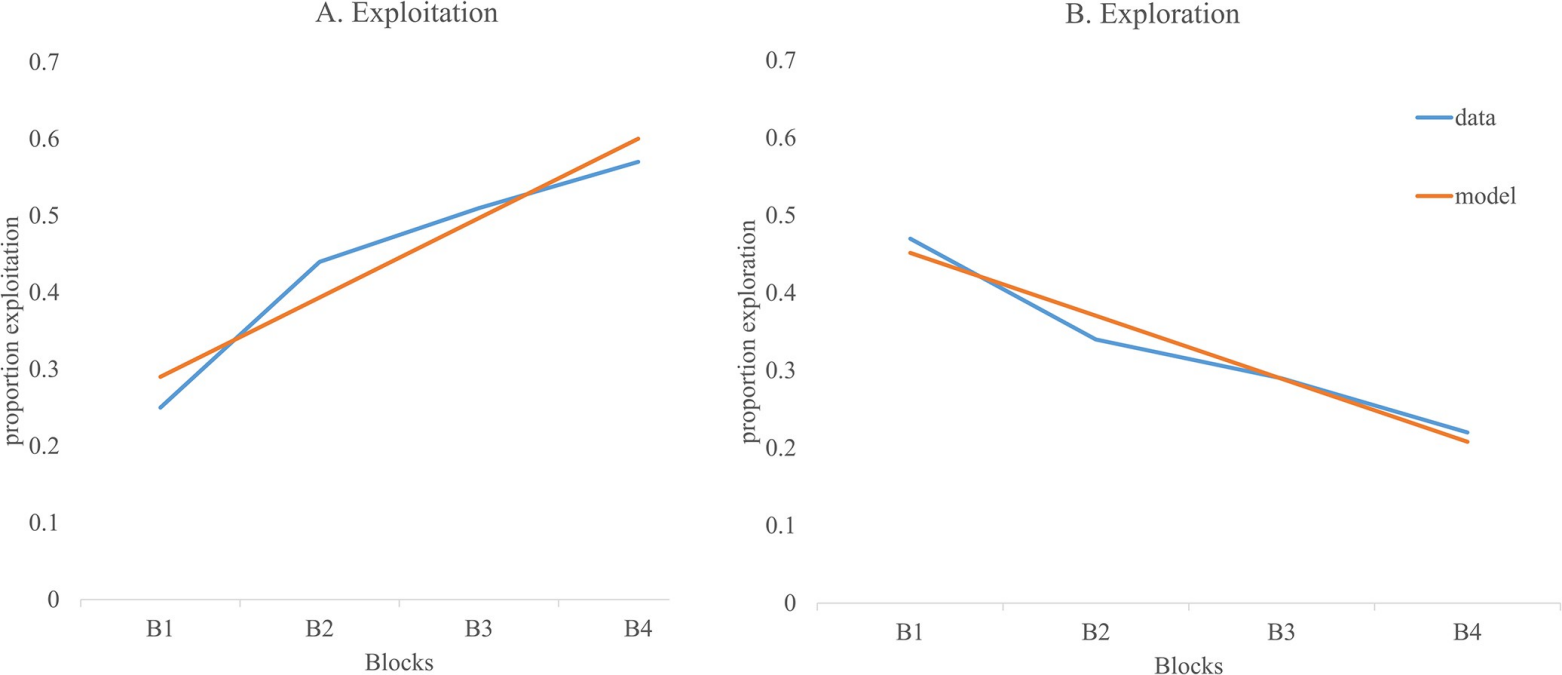
Proportion of exploitation and exploration on the PGT. The fitted models and means from the data (for comparison) are shown. (A) Model F from Table 3 is compared to the exploitation data means. Model F was fitted using data means. (B) Model E from Table 4 is compared to the exploration data means using the data means.

**Fig 7:**
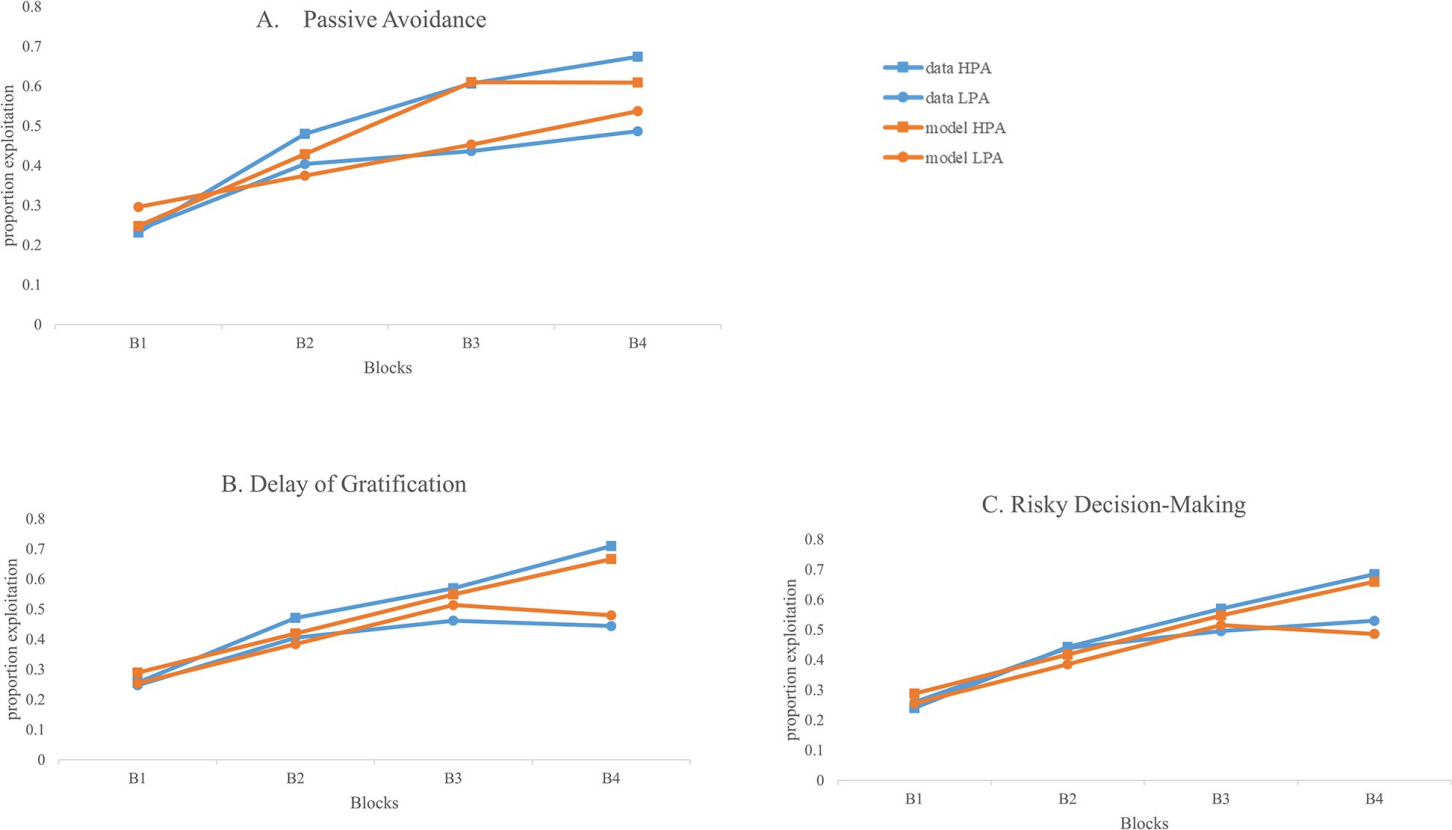
The prediction of exploitation using simpler decision-making tasks. (A) Model F from Table 3 is fitted for children scoring 1 SD above and below the mean on PA1. The sample exploitation means are shown for children above (n = 60) and below the mean (n = 74) on PA1 for comparison. (B) Model F from Table 3 is fitted for children scoring 1 SD above and below the mean on DoG. The sample exploitation means are shown for children above (n = 62) and below the mean (n = 71) on DoG for comparison. (C) Model F from Table 3 is fitted for children scoring 1 SD above and below the mean on RDloss. The sample exploitation means are shown for children above (n = 37) and below the mean (n = 96) on RDloss.

The main effect of block was significant, γ = .130, *p* < .001, with exploitation increasing an average of 13% every block (see Fig 6A). There was a significant effect for Block x PA1, γ = .369, *p* = .002. Follow up using simple slopes were calculated for low scores (low PA1: -1 SD) and high scores (high PA1: +1 SD) over the four blocks. The simple slopes for low PA1, *t* = 2.694, *p* = .008 and high PA1, *t* = 7.244, *p* < .001 were both significant. However, the high PA1 slope was steeper in comparison to the low PA1, suggesting that children with higher initial performance on passive avoidance increased their exploitation of the good cards on the PGT faster than those who had lower performance (Fig 7A). The Test x PA1 interaction was marginally significant, γ = -.603, *p* = .052. Follow up using simple slopes showed that there was no change in slope after the test for children with low scores on PA1, *t* = .114, *p* = .910. In contrast, children with high scores on the PA1 showed a decrease in slope following the awareness test, *t* = 2.804, *p* = .006. In other words, once the effect of block and the other predictors were controlled, being high on PA1 led to reduction in slope.

The Test x DoG interaction was significant, γ = .213, *p* = .018. Simple slopes were calculated for low scores (low DoG: -1 *SD*) and high scores (high DoG: +1 *SD*) on the delay of gratification task. The simple slope was significant for low DoG, *t* = 3.044, *p* = .003, with children who scored low on DoG showing a reduction in the exploitation slope after the awareness test; in contrast, the simple slope for children who had high scores on DoG showed no change in slope following the awareness test, *t* = .122, *p* = .903 (Fig 7B).

The Test Time x RD Loss interaction was also significant, γ = .408, *p* = .002. Simple slopes were calculated for low scores (low RD Loss: -1 *SD*) and high scores (high RD Loss: +1 *SD*) on the risky decision-making task. The simple slope for children who had low scores on the risky decision-making task was significant, *t* = 2.890, *p* = .005, with children who had low scores on risky decision-making showing a drop in the exploitation slope following the awareness test; in contrast, the change in slope for children who had high scores on risky decision-making was not significant, *t* = .309, *p* = .758 (Fig 7C).

### Prediction of exploration on the PGT

Preliminary analyses indicated that test time and age as fixed effects (as well as their interactions) did not contribute to the variance in exploration (all *p’s* > .1). Test time did not explain significant variance beyond what was already accounted for by block as a random variable, *σ*^*2*^ = .034, *p* = .161. As a result, test time was not added as a random or fixed variable. Similarly, age in months was not included in the analysis discussed below.

The addition of block in Model B (Table 4) significantly improved model fit, χ^2^ (3) = 146.723, *p* < .001, explaining 48.9% of within-person variation. The addition of main effects (Model C) improved model fit, χ^2^ (3) = 9.192, *p* = .028, explaining 2.1% of overall exploring. The addition of interactions with block (Model D) also significantly improved model fit, χ^2^ (3) = 21.680, *p* < .001, explaining an additional 3% of overall variation in exploring and 14.8% variation in changes of exploration across blocks. Given that there was only one significant interaction, the model was pruned (Model E). This model did not significantly differ from Model D, χ2 (2) = 1.469, *p* = .480. Model E was therefore retained as the final model.

**Table 4:** Summary of multilevel modeling for exploration as the dependent measure.

Effect	Model A	Model B	Model C	Model D	Model E	Model F	Model G
*Fixed Effects*							
Intercept	.329***	.452***	.451***	.451***	**.450** ***	**.303** **	.303**
Block		-.082***	-.082***	-.082***	**-.082** ***	**.029**	.029
Trans Err			.334*	.270	**.334** *	**.338** *	.340*
PA1			-.273	.218	**.225**	**.235**	.232
RD Loss			-.053*	-.205	**-.273** *	**-.266** *	-.263*
Block x Trans Err				.048			
Block x PA1				-.205**	**-.210** **	**-.176** **	-.176**
Block x RD Loss				-.052			
Aware Level						**-.007**	-.024
Block x Aware Level						**-.027** *	-.027*
Aware Level x Trans Err							.083
Aware Level x PA1							.104
Aware Level x RD Loss							.024
*Random Effects*							
Within-person	0.049***	0.025***	0.025***	0.025***	**0.025** ***	**.025** ***	.025***
Initial status	0.055***	0.068***	0.066***	0.064***	**0.065** ***	**.064** ***	.064***
Block effect		0.008***	0.008***	0.006***	**0.007** ***	**.006** ***	.006***
*Pseudo R* ^*2*^							
Within-person		.489	.489	.489	**.489**	**.489**	.489
Initial Status			.021	.051	**.047**	**.048**	.062
Block effect			.000	.148	**.132**	**.198**	.198
*Deviance*	131.739	-14.984	-24.176	-36.664	**-35.195**	**-43.978**	-44.652
AIC	137.739	-2.984	-6.176	-12.664	**-15.195**	**-19.978**	-14.652
BIC	150.569	22.676	32.314	38.656	**27.571**	**31.342**	48.498
Model test		A vs B	B vs C	D vs C	**E vs D**	**F vs E**	G vs F
Significance (χ^2^)		χ^2^ (3) = 146.723***	χ^2^ (3) = 9.192*	χ^2^ (3) = 21.680***	**χ**^**2**^ **(2) = 1.469**	**χ**^**2**^ (2) = 8.783*	χ^2^ (3) = 0.674
					**E vs C**		
					**χ**^**2**^ (1) = 11.019**		

Abbreviations: Trans Err = Transitivity Errors, PA1 = Passive Avoidance first half, D-prime score, RD Loss = Risky Decision Making, loss frame, Aware Level = Awareness Level. Two final models appear in bold.

^ŧ^ *p* < .1

*p < .05

**p < .01

***p < .001.

**Fig 8:**
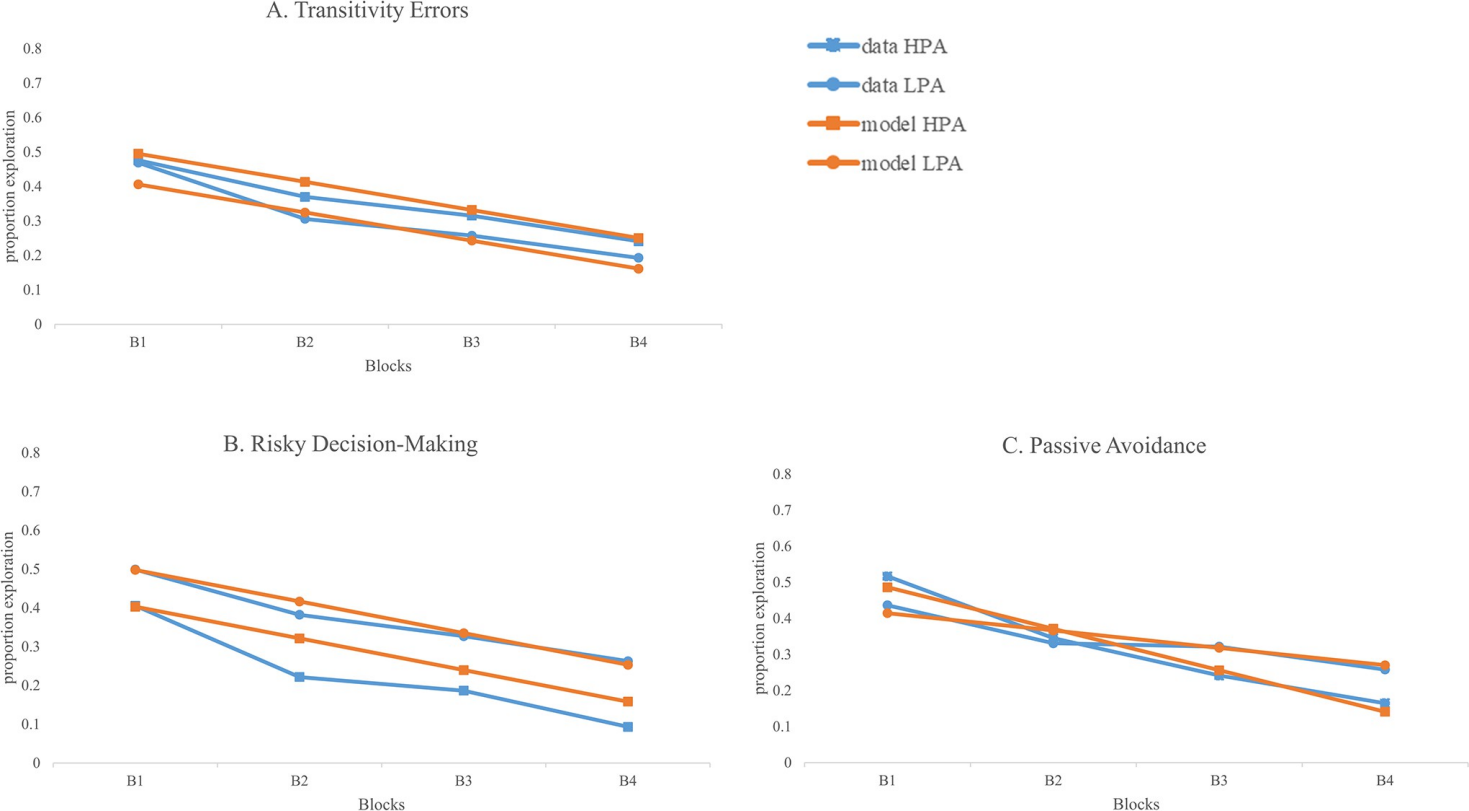
The prediction of exploration using simpler decision-making tasks. (A) Model E from Table 4 is fitted for children scoring 1 SD above and below the mean on Trans Err. The sample exploration means are shown for children above (n = 66) and below the mean (n = 68) on Trans Err for comparison. (B) Model E from Table 4 is fitted for children scoring 1 SD above and below the mean on RDloss. The sample exploration means are shown for children above (n = 37) and below the mean (n = 96) on PA1 for comparison. (C) Model E from Table 4 is fitted for children scoring 1 SD above and below the mean on PA1. The sample exploration means are shown for children above (n = 60) and below the mean (n = 74) on RDloss.

Inspecting the parameters indicated significant main effects of block, γ = —.082, *p* < .001, Trans Err, γ = .334, *p* = .046, and RD Loss, γ = —.273, *p* = .031 (Fig 8A and 8B). The parameters indicated that children showed a reduction of exploration by 8.2% for each block. Children who made a lot of transitivity errors also showed a high level of exploration while children who made advantageous decisions in the RD Loss task showed a lower amount of exploration overall. While the main effect of the passive avoidance task was not significant, there was a significant PA1 x Block interaction, γ = —.210, *p* = .001. Follow-up using simple slopes indicated that children who showed low performance on PA1 (-1SD) did not show a significant decrease in exploration across blocks, *t* = 0.089, *p* = .930 while those children who showed high performance on PA1 (+1SD) significantly decreased their exploration during the game, *t* = 2.776, *p* = .007 (Fig 8C).

### Comparison of exploitation and exploration slopes

In order to explore whether children shifted from exploration to exploitation during the course of the game, a simple slope comparison was run using the slopes from Model B of Table 3 (exploitation) and Model B of Table 4 (exploration). The slopes were found to be significantly different, *t* = 15.631, *p* < .001. More specifically, Holm-Bonferroni adjusted paired t-test for each block indicated that children showed significantly more exploration in comparison to exploitation in Block 1, *t* (133) = 5.377, *p* < .001, showed no significant difference in Block 2, *t* (133) = 1.882, *p* = .062, showed significantly higher exploitation in Block 3, *t* (133) = 3.833, *p* < .001 and Block 4, *t* (133) = 6.035, *p* < .001. In other words, children crossed over from favoring exploration to favoring exploitation by Block 3 (see Fig 6).

### Prediction of awareness on the PGT

The addition of block in Model B (Table 5) significantly improved model fit, χ^2^ (7) = 191.493, *p* < .001, explaining 66.7% of within person variation. The addition of main effects of age and delay of gratification (Model C) improved model fit, χ^2^ (2) = 24.299, *p* < .001, explaining 10.5% of between person variance in overall awareness. The addition of interactions with block and awareness type (Model D) did not significantly improve model fit, χ^2^ (5) = 8.924, *p* = .112. Inspection of parameters indicated that all the interactions except for the Type x Age interaction were non-significant. When the non-significant interactions were removed from the model (Model E), the model was significantly different from Model C, χ2 (1) = 6.152, *p* = .013. Model E was therefore retained as the final model.

**Table 5:** Summary of multilevel linear modeling for awareness as a dependent.

Effect	Model A	Model B	Model C	Model D	Model E
*Fixed Effects*					
Intercept	.1.024***	1.362***	1.367***	1.352***	**1.368** ***
Block		.026	.026	.057	**.026**
Aware Type		-.703***	-.703***	-.670***	**-.705** ***
Age			.027**	.015	**.017**
DoG			.450**	.510**	**.450** **
Block x Aware Type				-.078	
Block x Age				.005	
Block x DoG				-.188	
Aware Type x Age				.021*	**.023** *
Aware Type x DoG				.089	
*Random Effects*					
Within-person	.548***	.182***	.182***	.181***	**.182** ***
Initial status	.374***	.533***	.477***	.472***	**.471** ***
Block effect		.187***	.187***	.184***	**.187** ***
Type effect		.416***	.416***	.389***	**.389** ***
*Pseudo R* ^*2*^					
Within-person		.667	.667	.670	.667
Initial Status			.105	.115	**.116**
Block effect			.000	.019	**.000**
Type effect			.000	.064	**.065**
*Deviance*	1365.061	1173.568	1149.269	1140.345	1143.117
AIC	1371.061	1193.568	1173.269	1174.345	1169.117
BIC	1383.891	1236.334	1224.588	1247.048	1224.713
Model test		A vs B	B vs C	C vs D	**C vs E**
Significance (χ^2^)		χ^2^ (7) = 191.493***	χ^2^ (2) = 24.299***	χ^2^ (5) = 8.924	**χ**^**2**^ (1) = 6.152*

Abbreviations: Trans Err = Transitivity Errors, PA1 = Passive Avoidance first half, D-prime score, DoG = Delay of Gratification. Final model appears in bold.

*p < .05

**p < .01

***p < .001.

**Fig 9:**
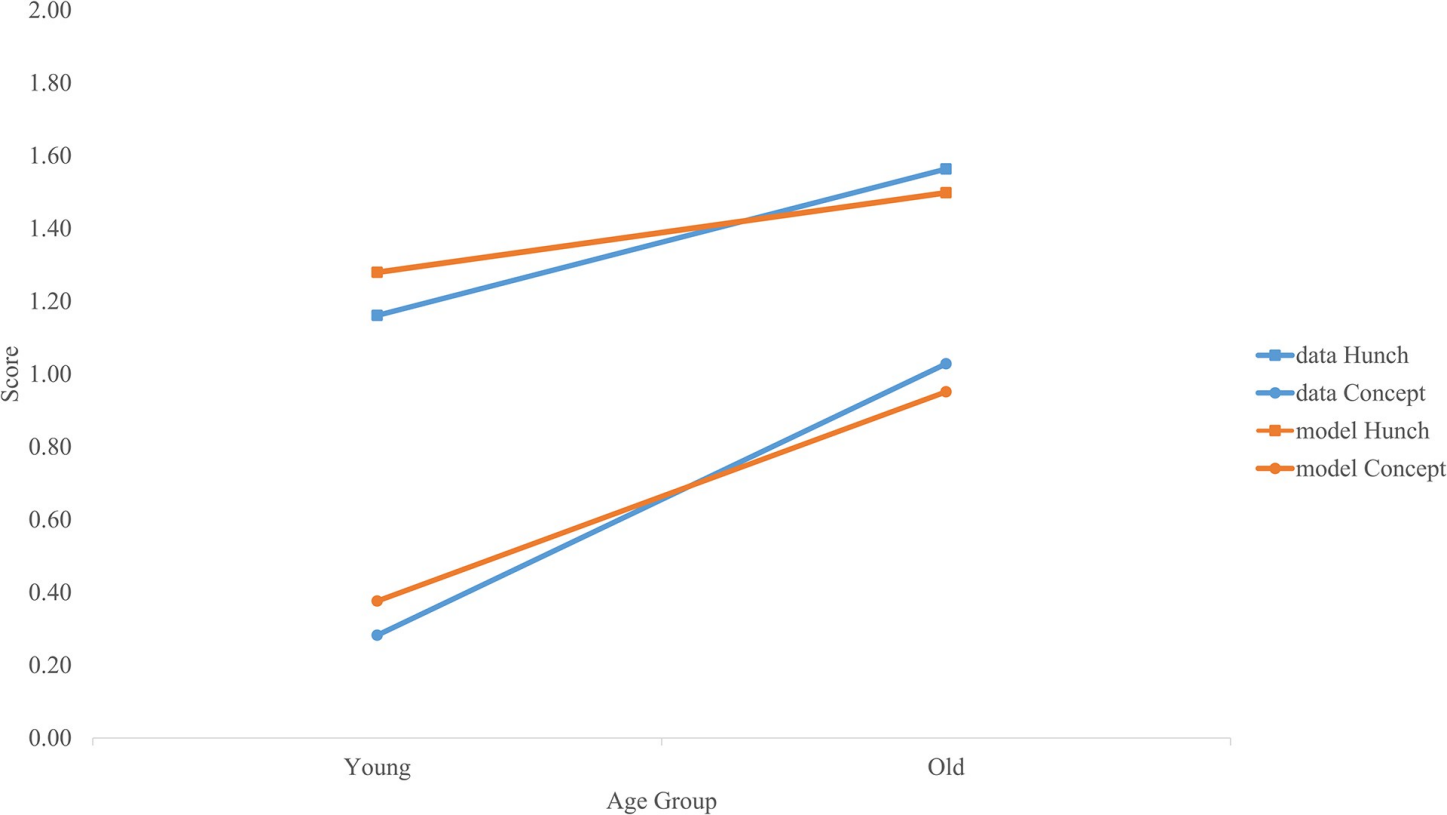
Prediction of awareness on the PGT. Model E of Table 5 was fitted for children who were 1 SD above and below the mean on age in months on hunch and conceptual awareness questions. For comparison, the awareness test scores for the sample are plotted for children who were older (n = 71) and younger (n = 62) than the sample mean (*M* = 49.99, *SD* = 7.27).

The parameters indicated a significant main effect of awareness type, γ = -.703, *p* < .001, where children had a higher score on the hunch questions compared to the conceptual awareness questions. There was also a main effect of delay of gratification, γ = .450, *p* = .005, where children with higher scores on delay of gratification also had a higher level of awareness on the PGT. Finally, there was a significant Type x Age interaction, γ = .023, *p* = .013. Follow up indicated no significant age differences for the hunch questions, *t* = 1.845, *p* = .067 while there was a significant age difference for conceptual awareness questions, *t* = 4.043, *p* < .001 (Fig 9).

### Contribution of awareness to exploitation and exploration

For exploitation, the addition of awareness and its interaction terms in Model G (Table 3) significantly improved model fit, χ2 (3) = 29.275, *p* < .001, explaining an additional 1.6% of the variance in overall exploitation, an additional 13.1% of variance across block, and 8.4% of variance in change following the awareness test. The addition of interactions of the decision-making tasks with awareness in Model H did not significantly improve the model fit, χ2 (3) = .888, *p* > .1. Model G was thus retained as the final model.

**Fig 10:**
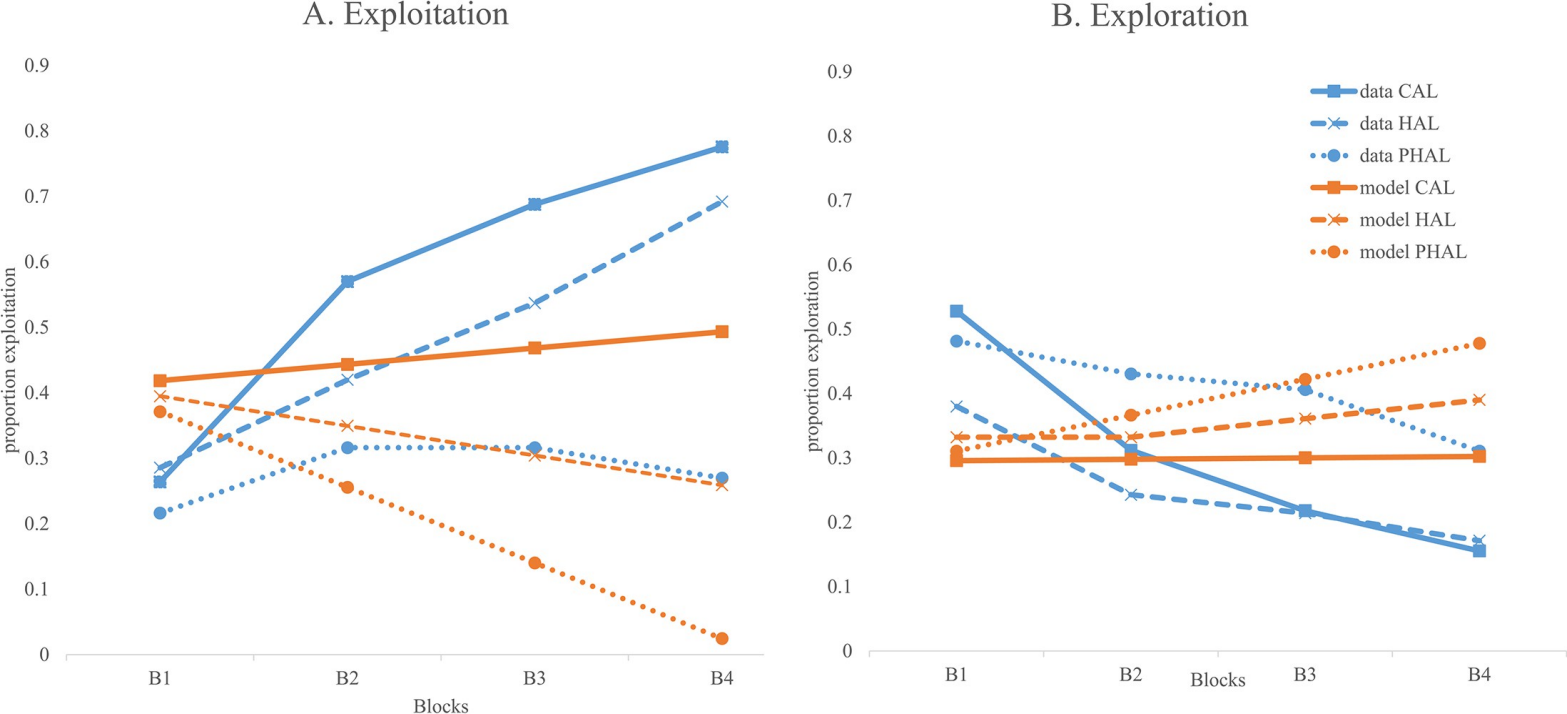
Prediction of exploitation and exploration using decision-making tasks and awareness levels. (A) Model G from Table 3 is fitted for exploitation as a function of awareness level (-1, 0, and 1). For comparison, group exploitation means for children classified in the conceptual level group (n = 50), the hunch level group (n = 35) and pre-hunch level (n = 49) are shown. (B) Model F of Table 4 is fitted for exploration as a function of awareness level. For comparison, group exploitation means for children classified in the conceptual level group, the hunch level group and pre-hunch level are shown. Abbreviations: PGT = Preschool Gambling task, CAL = conceptual awareness level, HAL = hunch awareness level, and PHAL = pre-hunch awareness.

Inspection of parameters indicated a significant interaction of Block X Aware Level, γ = —.070, *p* = .001. No other effect with awareness was significant. Follow-up using simple slopes indicated that children who had a higher awareness level in comparison to those who had a lower awareness level exploited more from Block 2, *t* = 3.490, *p* < .001 to Block 4, *t* = 4.091, *p* < .001 (Fig 10A). Note that the previously significant Test x DoG interaction became non-significant once the awareness terms were entered. Mediation analysis indicated that the association of DoG performance with the last block of the PGT was mediated by awareness level at the end of Block 3 (*B* = .016; *CI* = .01 to .02). Furthermore, once awareness was added to the model, DoG became non-significant (*B* = .01, *t* (132) = 1.78, *p* = .077), suggesting better performance on the last block of the PGT by high DoG performance was due mostly to the abilities involved in better awareness level. The Block x PA1 remained significant.

For exploration, the addition of awareness and its interaction terms in Model F (Table 4) significantly improved model fit, χ2 (2) = 8.783, *p* = .012, explaining an additional 6.6% of the variance in exploration. The addition of interaction of decision-making tasks with aware level (Model G) did not significantly improve model fit, χ2 (3) = 0.674, *p* = .879. Model F was thus retained as the final model.

Inspection of parameters indicated a significant interaction of Block x Aware Level, γ = -.027, *p* = .016. Follow-up using simple slopes indicated that children with higher awareness in comparison to children with lower awareness showed less exploration from Block 3, *t* = 2.314, *p* = .022 to Block 4, *t* = 2.835, *p* = .005 (Fig 10B).

## Discussion

The current findings provide support for the extension of an integrated decision-making framework [10, 11, 15] to very young children. While there is support for distinct stages in decision-making for older children and adults, there is comparatively little work done in early childhood. The current study replicates the findings of Garon and English [21], indicating that young children show a higher degree of exploration early in the PGT and switch to a higher degree of exploitation later when they have accumulated knowledge (Fig 6). In addition, the findings indicate that, consistent with the framework, the degree of exploration and exploitation varies according to the awareness level children have reached by the end of the third block. Those with higher awareness show a higher level of exploitation in the last three blocks and a lower level of exploration in the last two blocks. Additionally, higher awareness explained additional variance beyond that explained by the decision-making tasks. Finally, while we found support for different mechanisms mediating the two stages of decision-making, some of our predictions were not supported. We discuss these findings in more detail below.

### Strategy use across stages and awareness level

The findings from the current study replicated findings of Garon and English [21]. On average, preschoolers showed increasing exploitation and decreasing exploration across the blocks of the PGT (see Fig 6). This suggests that children were initially switching back and forth between decks, collecting knowledge via feedback. It also suggests that they reduced their exploration gradually as they became more certain about the contingencies of the PGT.

Our findings, however, indicate that there is a lot of individual variability in children’s strategy use. One of these sources of variability is awareness level. As Fig 10 demonstrates, there is a difference in strategy use in children with different levels of awareness. The data lines (blue) represent the average exploitation and exploration of children categorized in the pre-hunch, hunch, and conceptual awareness stages. For instance, when comparing Fig 10A and 10B, it appears as though children who stayed in the pre-hunch stage (line with circles) maintained an exploration rate that was higher than exploitation. This suggests that children in this group were in a state of uncertainty throughout the game, not showing a preference for either deck. In sharp contrast, children who had reached the hunch (line with x’s) or conceptual (line with squares) stage showed a robust increase in exploitation combined with a decrease in exploration.

The difference between children in the hunch and conceptual group are subtle, but also interesting. Note that the two groups do not show many differences in the rate of exploration except for a higher initial rate of exploration for children in the conceptual group. However, children in the conceptual group develop a preference for the advantageous deck by the second block, reaching an exploitation rate of 60% and increasing to over 80% by the end of the game (Fig 10A). In contrast, children in the hunch group show a slower rate of increase, initially beginning at a higher exploitation rate and gradually increasing to above 70% (Fig 10A). The strategy pattern of the children in the conceptual stage indicates a more effortful, explicit strategy from the beginning. They explore the decks more in the beginning, perhaps forming a stronger representation of the game as a result. In sum, the results from the last set of analyses supports the idea that awareness of the PGT has an impact on decision-making above that which is accounted for by other decision-making, skills such as learning from feedback and the ability to weigh gains and losses in making choices.

Finally, another interesting observation when looking at Fig 10 is the difference between the lines from the fitted model (orange) versus the data lines (blue). Fig 10A was fitted using Model G of Table 3 and Fig 10B was fitted using Model F of Table 4. For exploitation, the model lines do not show as much continuous elevation as the data lines. Since the values for decision-making predictors were set at the mean (or “0” as the variables were centered) when the model was fit, this difference illustrates the different contributions of awareness versus the other decision-making tasks to the prediction of performance on the PGT. While awareness seems to influence a gradual increase in exploitation choice and shifts in choice over the course of the blocks, feedback learning as assessed by PA1 (Fig 7A) seems to influence choice behavior in a more pronounced manner. Note that low awareness seems to create a reduction of exploitation across blocks (orange lines with circles) whereas high awareness appears to produce a small increase in exploitation across blocks (orange line with squares). Feedback learning as assessed by PA1, on the other hand, appears to lead to a steeper increase in exploitation and no decrease in exploitation for low feedback learning (Fig 7A). Correspondingly, awareness as predicted by the model (Fig 10B) appears to differentially influence exploration in comparison to feedback learning as assessed by PA1 (Fig 8C). While low awareness (orange lines with circles, Fig 10B) appears to be associated with an increase in exploration, high awareness (orange lines with squares, Fig 10B) shows no impact on exploration. In contrast, high feedback learning (orange lines with squares, Fig 8C) appears to be associated with an overall reduction in exploration across blocks. It may be that what you do with your awareness of the game depends on which way your implicit learning is pulling you. We turn to this issue next.

### The influence of learning on strategy use

Our findings support a role for learning from feedback during most of the game. Performance on the initial portion of the passive avoidance (PA) task was found to explain individual differences in performance throughout the game. Fig 7A shows that children who were one standard deviation above the mean on the first part of PA1 showed a steeper increase in exploitation in comparison to children who were one standard deviation below the mean. These findings support the idea that better ability to learn from loss and win feedback leads to an increase in exploitation on the PGT. Furthermore, this difference in exploitation reflects a change in choice across blocks rather than just an overall increase in exploitation; this indicates a continuous update in children’s representation of the game. The corresponding change in exploration across the game of these high scoring PA1 children additionally indicates a reduction in uncertainty about the game. Whereas children who were low on PA1 showed no change in exploration across blocks, children high on PA1 showed a significant decrease in exploration (Fig 8C).

While this illustrates exploration and exploitation as a function of PA1 performance, it does not explain the trend of a decrease in the exploitation slope following the awareness test found for children with higher PA performance. This finding was puzzling, particularly since we hypothesized that children with higher PA performance would show a higher awareness level. However, S1 Fig suggests an explanation for this finding. The group of children in the high PA1 group have approximately one quarter (25.71%) who can be classified as pre-hunch. These children appear to show a drop in exploitation from Block 3 to 4. In fact, when the awareness level is added to the analysis (Table 3, Model G), the trend disappears. This suggests that the decrease in exploitation after the awareness test is due to this group of children.

Another interesting finding is the lack of association between PA performance and awareness on the PGT. Why does higher awareness not follow from an advantage in learning from feedback? The answer may lie in the partial dissociation between implicit and explicit learning [59]. In their review of the literature, Helie and Sun [59] argued that implicit and explicit learning occurred in parallel but influenced behavior in different ways. Whereas implicit learning leads to a slow change in behavior, explicit learning tends to have a more dramatic influence on behavior, particularly when explicit knowledge is gained through interaction of the explicit with implicit processes [59]. The current findings suggest a dissociation between implicit and explicit knowledge in at least some children.

In fact, there may be differences in the population in how implicit and explicit knowledge are associated to one another and in turn how these different forms of knowledge are associated with choice. For example, Delgado and colleagues [60] found that children from higher SES backgrounds showed an association between anticipatory heart rate (HR) deceleration in response to the disadvantageous deck and higher awareness; furthermore, HR deceleration and awareness was associated with better performance on the Children’s Gambling task (CGT). In contrast, anticipatory HR *acceleration* to the disadvantageous deck was associated with higher awareness in the low SES group; neither HR nor awareness were significantly associated with choice in this group. Interestingly, there was no difference in awareness on the CGT between these two groups of children, suggesting performance differences were not due to cognitive differences [22]. One possible explanation for these findings is that children in this group have difficulty forming an implicit representation and disregard their explicit information in making a choice. Another possibility is that they have difficulty translating their implicit representation into explicit information, creating a representation that carries less motivation to choose advantageously. Interestingly, recent research indicates that HR deceleration may be associated with becoming aware of a sensory percept that was initially below participants’ level of awareness [61]. This is particularly interesting given the previous finding of a positive association between awareness and performance on the CGT in a group of middle to high SES children, but no association between awareness and performance in low SES children [22, 60].

If implicit and explicit knowledge differ, what determines choice? As our discussion on awareness indicates, both types of knowledge contributed to choice in different ways in the current study. For example, S1 Fig shows that children in the low and high PA1 groups showed very different patterns of choice depending on their level of awareness at the end of the third block. Those children who were high on PA1, but low on awareness (pre-hunch) showed no significant increase in exploitation (see S1 Table). In contrast, those children who were low on PA1 but high on awareness level (hunch or conceptual), showed an improvement across the four blocks, but did not seem to do as well as those children with both high PA1 and a high awareness level (see S1 Table with accompanying supplemental analyses). In other words, learning from feedback is important, but the best performance appears to require an interaction of implicit and explicit processes. In support of this, Hartley and colleagues [62] argued that for children, an ability to use a mental model to guide decisions may require the ability to construct an explicit representation of environmental contingencies.

### Strategy use when options are known

There were three tasks in the current study that investigated decisions made when options are known. While performance on the three tasks did predict performance in the last half of the PGT, there were unexpected findings that provided some insight into the mechanisms of early decision-making in young children.

The simplest task involved making repeated choices between paired toys. We explored errors of transitivity as an assessment of inconsistency of choice. The transitivity task was associated with exploration but not exploitation on the PGT. The literature indicates that exploration is associated with knowledge status and level of certainty [63, 64]. Folke et al. [63] found that individuals who reported more confidence in their decisions when making choices about foods had significantly fewer errors of transitivity, suggesting that more consistent choices reflect higher certainty. The lack of association of this task with exploitation highlights the idea that exploitation and exploration are mediated by different processes. Blanchard et al. [64] argued that an anterior cingulate and insula network was important for exploration, while a ventromedial network played an important role in updating expectations, and therefore, in exploitation. The findings also suggest that being more consistent does not necessarily lead to better choices (i.e., higher exploitation) on the PGT. In other words, children who had lower errors of transitivity increased their number of choices on one deck, but not necessarily the good deck.

The DoG task is the most popular decision-making task used in the developmental literature [4]. The version of the task used in the current study combined the elements of choice and waiting, being partly inspired by the animal literature [51]. As expected, it predicted better performance in the latter portion of the PGT, which supported the hypothesis that children who chose to sustain delay in the DoG for a larger reward were the same children who made more advantageous choices once they had knowledge of the game. However, unexpectedly, better performance on the DoG was associated with higher awareness on the first awareness test. Given that the DoG was not theorized to be linked to learning from contingencies, this was another puzzling finding.

One possible reason for this association is that performance on the DoG and reported awareness on the PGT depends on common processes. A likely contender for a common process is the ability to reflect on bodily responses (e.g., increased heart rate) evoked by environmental cues. Past research has indicated that having preschool children reflect upon their experience with a preschool version of the IGT improve their subsequent performance on the task [17]. Moreover, children who can reflect on bodily responses and emotions associated with receiving a larger future reward may increase their motivation to delay. Along this line, Bulley et al. [65] argued that imagining the future improves DoG performance because it increases the value representation of the future reward. In other words, it may be that the ability to reflect upon feeling states (current and anticipated) enables children to not only delay longer, but also become explicitly aware of their feelings about the decks. Furthermore, it is quite possible that the association of the DoG with the last PGT block was due to the ability to reflect on feeling states rather than the ability to choose between two options. The results of the mediational analysis support this idea, indicating that the association between DoG performance and exploitation on the PGT was fully mediated by a child’s awareness level just before they made choices on the last block.

Along this line, recall that children in the low SES group chose more disadvantageous cards despite a similar awareness level to the other SES group [22, 60]. Delgado et al. [22] hypothesized that it could be due to a dissociation between awareness and performance on the CGT in children from the low SES group. As noted in the last section, they have found differences in association between HR deceleration and awareness on the CGT in these two SES groups [60]. Delgado et al. [60] speculated that it may have something to do with higher stressors in the early history of children with lower SES backgrounds, resulting in an attentional style that is more defensive in nature. They also hypothesized that this may lead to a difference in valuing short- versus long-term options. In other words, rather being a lack of association between awareness and performance, the SES difference in performance may due to differences in valuation of the two decks. In fact, recent research indicates a correlation between choosing the immediate alternative and school outcome in a sample of low SES children, suggesting that depending on your life context, the immediate option may be more adaptive [66].

We had expected that both loss and gain domains of risky decision-making would be associated with PGT performance. However, only performance on the loss domain was found to be associated with performance on the last block of the PGT. A possible reason for this is that the loss domain may have been more salient to children. Another unexpected finding was that performance on the risky decision-making task showed a different pattern of association than the DoG did with the PGT. First, performance on the RD Loss task was not associated with awareness level on the PGT. Second, performance on this task was inversely associated with exploration on the PGT.

What could have accounted for this different pattern of results on these two explicit decision-making tasks? One likely reason is that while DoG relies on the comparison of values associated with different time points, risky decision-making relies on the comparison of values associated with different probabilities. Hence, while both tasks are explicit and both involve comparison of values, the subjective values must be adjusted using either time or probability [67]. It is likely that the two comparisons rely on different processes [68, 69]. For instance, Luhmann [69] argued that temporal choices would rely not only on comparing values, but also imagining one’s own subjective experience in waiting. Decisions on the RD Loss task are less reliant on imagination and reflection as they involve a comparison of values that differ in probability but are nonetheless immediately available once a choice is made.

Furthermore, part of the decision process in risky decision-making involves an individual’s tolerance for uncertainty [70]. This idea is supported by the finding of an association between the RD task and exploration (see Table 1). The ability to tolerate uncertainty shows a great deal of individual variability [71]. Since both decks on the PGT are associated with uncertainty and trials involve choosing one of the two decks, individuals with high intolerance for uncertainty might never develop a deck preference; rather uncertainty might overshadow the ability to appreciate differences in the magnitude of gains and losses associated with both decks. Kornilova et al. [71] found that high intolerance for uncertainty was associated with increasing shifting after loss in decks. Similarly, the uncertainty involved in the risky decision-making task might make choices more difficult for children who find uncertainty aversive, particularly in the loss frame. The lack of association between RD Loss and DoG tasks supports the idea that the common variance between RD Loss and exploration is the result of difficulty making choices in uncertain situations. In other words, children who had difficulty making good choices in the risky decision task did not necessarily have difficulty making good choices in the DoG, a task that does not involve uncertainty.

### Limitations and future directions

The current study indicates a more complicated picture of decision-making than just a simple division of decisions made when options are not known versus when they are known. While the current study indicates different processes involved in decisions under risk, it provides less information on the learning portion of decision-making. One limitation of this study is the inclusion of only one task for assessing decisions under ambiguity. Future research should investigate this early process by using other tasks that involve learning from feedback. There are some possibilities from the literature. For instance, probabilistic learning tasks, which involve two or more options varying in the probability of receiving a reinforcement, have been found to show age differences in older children [72]. Similarly, reversal learning, which involves reversing contingencies once an option is consistently chosen, would also be another option as versions of these have been adapted for preschoolers [73]. Another limitation of the current study is the failure to use physiological measures such heart rate or skin conductance response. Future research using physiological would be valuable to assess the association of these measures with awareness and performance on the PGT. It would also be interesting to look at the association with other tasks such as passive avoidance and delay of gratification, which we hypothesized would be associated with the valuation process and choice.

Another limitation is the use of only 40 trials on the PGT. It would be interesting to look at the association of the delay of gratification task and risky decision task on later trials. It may be that later blocks would show a stronger association with these tasks that assess the ability to make decisions once contingencies are known.

One issue that was not addressed in this paper was the transition from exploration to exploitation. We know that the transition to exploitation depends in part on whether learning takes place [11], but it is also dependent on uncertainty. Research indicates that the ability to learn from feedback and uncertainty are mediated by related, but partially dissociable mechanisms [74]. Children who can learn contingencies but remain uncertain may not be able to transition to exploitation. This may have been the case for children who had a high score on the passive avoidance task, but who had low awareness. It is also unclear whether the two strategies (exploitation vs. exploration) translate to important differences in real life. Future research should investigate whether these two strategies are associated with different long-term outcomes. Another avenue for research in this area is treatment to improve this transition in individuals who have high uncertainty intolerance.

Finally, our sample was homogeneous, being made up primarily of children from middle-class white families. As noted earlier, recent findings indicate that children from lower SES backgrounds may not approach affective decision-making tasks in the same manner as children from middle-class backgrounds [22, 60]. As such, our findings may not generalize to children from more diverse demographics. It will be interesting in the future to explore the use of exploitation and exploration strategies in children from more diverse SES and cultural backgrounds.

In conclusion, the current findings indicate that skills underlying various aspects of decision-making already show development in preschool. The current battery holds promise not just for assessment, but also for investigating this skill set in early childhood. Despite its potential for moving the field forward, there is currently a paucity of tasks to assess decision-making in early childhood. The most used task, the delay of gratification task, assesses only one aspect of decision-making. On the other hand, variants of the IGT assess co-ordination of various skills, such as learning from feedback, keeping track of contingencies that vary over time, development of a preference, and using accumulated knowledge to make decisions. One drawback of this task is the number of trials that must be administered to children. Furthermore, while doing well at this task suggests an ability to co-ordinate a variety of skills to make good decisions, doing poorly makes it unclear which skill is affected. Having a variety of tasks for this age range provides more flexibility for both clinicians and researchers.

The findings of the current study suggest that, even during the preschool period, there is much individual variability in these decision-making skills. Moreover, the findings suggest that the PGT involves multiple skills, and that awareness of the game contingencies explains additional variance in individual differences. The findings also suggest that the use of exploration and exploitation measures may be a more informative metric of the child’s performance on the PGT. Rather than providing one score, such as number of good choices, it provides a glimpse into how an individual adapts to ambiguity in their environment, a key problem in many psychiatric disorders [75]. As such, these measures have the potential to improve assessment of early developmental problems.

## Supporting information

S1 Fig Proportion of exploitation and exploration on the PGT as a function of pa1 performance and awareness level. PGT = Preschool Gambling task, PA = Passive Avoidance. PA groups were created by dividing the sample based on performance on the passive avoidance task and awareness level at the end of Block 3 on the PGT. Low PA: pre-hunch (n = 31), hunch (n = 19), conceptual (n = 24); High PA:: pre-hunch (n = 18), hunch (n = 16), conceptual (n = 26).
(TIF)

S1 Table Summary of differences in exploitation and exploration slopes as a function of awareness level and passive avoidance score. Abbreviations: PGT = Preschool Gambling task, PA = Passive Avoidance.
(DOCX)

S1 Dataset Decompose data set. (PDF)
