# Supplementary figures and images for: Decomposing decision-making in preschoolers: Making decisions under ambiguity versus risk
Source: PLoS One. 2024 Sep 30;19(9):e0311295. doi: 10.1371/journal.pone.0311295 (PMC11441697; doi:10.1371/journal.pone.0311295)

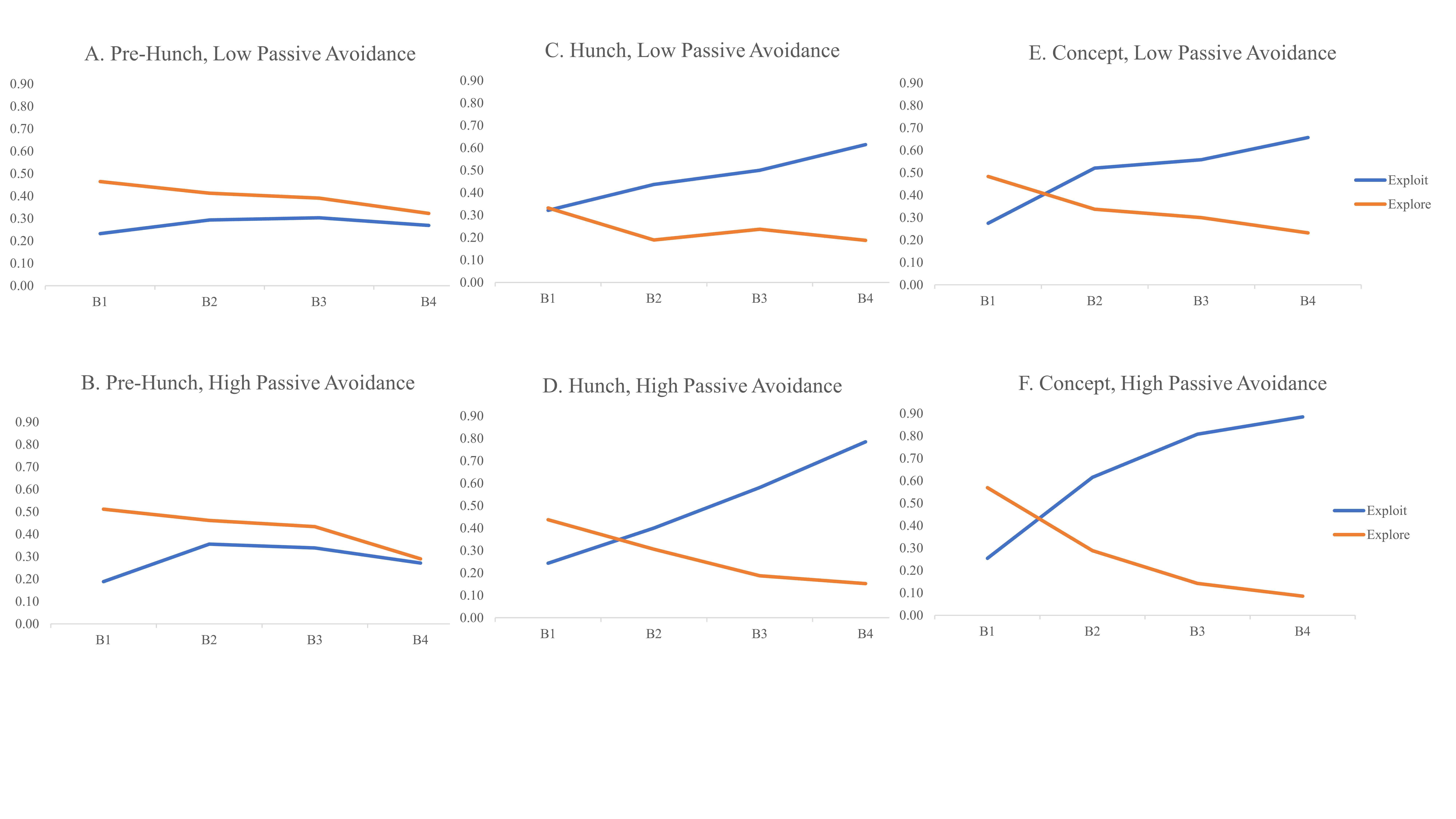

Supplement: S1 Fig — PGT = Preschool Gambling task, PA = Passive Avoidance. PA groups were created by dividing the sample based on performance on the passive avoidance task and awareness level at the end of Block 3 on the PGT. Low PA: pre-hunch (n = 31), hunch (n = 19), conceptual (n = 24); High PA:: pre-hunch (n = 18), hunch (n = 16), conceptual (n = 26). (TIF) [file pone.0311295.s001.TIF]
