# Supplementary material for: Decomposing decision-making in preschoolers: Making decisions under ambiguity versus risk
Source: PLoS One. 2024 Sep 30;19(9):e0311295. doi: 10.1371/journal.pone.0311295 (PMC11441697; doi:10.1371/journal.pone.0311295)
Supplement: S1 Table — Abbreviations: PGT = Preschool Gambling task, PA = Passive Avoidance. (DOCX) [file pone.0311295.s002.docx]

| **Table S1. Summary of differences in exploitation and exploration slopes as a function of awareness level and passive avoidance score**. Abbreviations: PGT = Preschool Gambling task, PA= Passive Avoidance. | | | | | | |
| --- | --- | --- | --- | --- | --- | --- |
|  | | | | | | |
| Slope Parameter | Estimate | Standard error | df | t-value | p-value | Holm-Bonferroni Adjusted p-values |
| PGT Exploitation | | | | | | |
| Pre-Hunch, PA low | .012 | .021 | 93 | .578 | .564 | .612 |
| Pre-Hunch, PA high | .023 | .022 | 54 | 1.034 | .306 | .612 |
| Hunch, PA low | .094 | .035 | 57 | 2.689 | .009 | .036* |
| Hunch, PA high | .180 | .033 | 48 | 4.459 | <.001 | <.001* |
| Concept, PA low | .118 | .027 | 72 | 4.462 | <.001 | <.001* |
| Concept, PA high | .208 | .019 | 78 | 11.053 | <.001 | <.001* |
| PGT Exploration | | | | | | |
| Pre-Hunch, PA low | -.045 | .015 | 93 | -3.047 | .003 | .015* |
| Pre-Hunch, PA high | -.069 | .017 | 54 | -4.058 | <.001 | <.001* |
| Hunch, PA low | -.039 | .017 | 57 | -2.239 | .029 | .087 |
| Hunch, PA high | -.097 | .024 | 48 | -4.117 | <.001 | <.001* |
| Concept, PA low | -.079 | .020 | 72 | -2.847 | . <.001 | . <.001* |
| Concept, PA high | -.160 | .015 | 78 | -10.555 | <.001 | <.001* |
|  |  |  |  |  |  |  |

To follow up the individual impact of feedback learning as measured by passive avoidance versus awareness level, twelve multilevel models were calculated to examine the slope of exploitation and exploration on the PGT. Block was entered as a fixed variable with intercept entered as a random variable. Children were divided into six groups based on their awareness level (pre-hunch, hunch, and conceptual) and scoring below (low PA1) or above (high PA1) the mean of the first half of passive avoidance. Table S1 summarizes the findings of the block effects, with Holm-Bonferroni adjusted p-values. As can be seen, five groups of children showed a significant decrease in exploration across the four blocks. The only group showing no significant decrease was the children in the hunch group with low scores on the first half of the passive avoidance task. Moreover, this group approached significance (*p* = .087).

However, in terms of exploitation, the picture is quite different (see Figure S1). Children in the pre-hunch stage, regardless of PA1 scores did not show increasing exploitation across blocks. In other words, the children in these groups were not showing an increasing preference for the advantageous deck (see Figure S1). In contrast, children who were either in hunch or conceptual awareness levels, regardless of PA1 scores showed significantly increasing preference for the advantageous deck.

Comparison of the slopes for children scoring higher on PA1 versus children scoring lower on PA1 indicated a steeper rise in exploitation for both the hunch groups, *t* (103) =2.498, *p* = .014 and children in conceptual groups, *t* (148) = 2.106, *p* = .037. The slopes did not differ for children in the pre-hunch groups, *t* (155) = .260, *p* = .795. For exploration, comparison of the slopes indicated that children scoring higher on the PA1 did not show a significantly greater decrease in exploration in comparison to children scoring lower on the PA1 for both the children in the pre-hunch groups, *t* (155 ) = 1.5120, *p* = .133 and hunch groups, *t* (103) = 1.225, *p* = .223. However, children who had higher scores on the PA1 decreased exploration significantly more than children who had lower PA1 scores in the conceptual awareness groups, *t* (148) = 4.577, *p* < .001.

In sum, having a high level of feedback learning is not enough if you have not reached at least a hunch level of awareness. Conversely, the differences between children having high versus low PA1 scores are more pronounced in the group of children who have the highest level of awareness.
