# Supplementary material for: Decomposing decision-making in preschoolers: Making decisions under ambiguity versus risk
Source: PLoS One. 2024 Sep 30;19(9):e0311295. doi: 10.1371/journal.pone.0311295 (PMC11441697; doi:10.1371/journal.pone.0311295)
Supplement: S1 Dataset — (PDF) [file pone.0311295.s003.pdf]

ID17_18	agemth	Know1	Know2	Why1	Why2	p_PA_1c	p_PA_2c	p_DoGt	p_EVlss	p_EVgn	p_intrtot	agmt_c	pintrtot_c	pPA_1_c	pPA_2_c	pDoG_c	pEVlss_c	pEVgn_c	exploit.1	exploit.2	exploit.3	exploit.4	explore.1	explore.2	explore.3	explore.4
1	40.00	2.00	2.00	1.00	0.00	0.57	0.52	0.17	0.40	0.40	0.40	-9.99	0.20	-0.06	-0.13	-0.35	-0.03	-0.10	0.00	0.10	0.10	0.00	0.80	0.70	0.80	0.78
2	47.00	1.00	0.00	0.00	0.00	0.59	0.64	0.83	0.40	0.40	0.05	-2.99	-0.15	-0.04	-0.01	0.32	-0.03	-0.10	0.10	0.40	0.10	0.11	0.60	0.40	0.80	0.78
3	54.00	2.00	2.00	1.00	2.00	0.51	0.60	0.33	0.40	1.00	0.20	4.01	0.00	-0.12	-0.04	-0.18	-0.03	0.50	0.10	0.10	0.00	0.33	0.80	0.50	0.60	0.67
4	54.00	0.00	1.00	1.00	1.00	0.33	0.64	0.08	0.40	0.40	0.00	4.01	-0.20	-0.30	-0.01	-0.43	-0.03	-0.10	0.00	0.30	0.00	0.67	0.50	0.60	1.00	0.33
5	56.00	0.00	2.00	0.00	0.00	0.82	0.95	0.58	0.60	0.40	0.40	6.01	0.20	0.19	0.30	0.07	0.17	-0.10	0.00	0.70	1.00	1.00	0.00	0.10	0.00	0.00
6	56.00	2.00	1.00	2.00	0.00	0.57	0.52	0.58	0.60	0.80	0.10	6.01	-0.10	-0.06	-0.13	0.07	0.17	0.30	0.60	1.00	1.00	1.00	0.20	0.00	0.00	0.00
7	56.00	0.00	0.00	0.00	0.00	0.95	1.00	0.33	0.20	0.60	0.00	6.01	-0.20	0.33	0.35	-0.18	-0.23	0.10	0.20	0.00	0.00	0.00	0.60	0.60	0.60	0.44
8	58.00	2.00	2.00	0.00	0.00	0.79	0.84	0.92	0.60	1.00	0.20	8.01	0.00	0.16	0.19	0.40	0.17	0.50	0.20	0.20	0.30	0.22	0.80	0.60	0.40	0.56
9	47.00	2.00	2.00	0.00	0.00	0.69	0.80	0.00	0.60	0.40	0.05	-2.99	-0.15	0.06	0.16	-0.51	0.17	-0.10	0.00	0.10	1.00	1.00	0.30	0.10	0.00	0.00
11	54.00	2.00	2.00	0.00	2.00	0.79	0.58	0.25	0.60	0.60	0.05	4.01	-0.15	0.16	-0.06	-0.26	0.17	0.10	0.20	0.40	1.00	1.00	0.40	0.50	0.00	0.00
12	55.00	0.00	2.00	0.00	2.00	0.60	0.32	0.25	0.60	1.00	0.20	5.01	0.00	-0.03	-0.32	-0.26	0.17	0.50	0.40	0.60	0.20	0.22	0.40	0.10	0.70	0.67
14	51.00	2.00	2.00	2.00	2.00	0.51	0.00	0.58	0.20	0.40	0.05	1.01	-0.15	-0.12	-0.48	0.07	-0.23	-0.10	0.40	1.00	0.50	0.00	0.20	0.00	0.10	0.11
15	59.00	2.00	2.00	0.00	0.00	0.54	0.74	0.25	0.20	0.40	0.00	9.01	-0.20	-0.09	0.10	-0.26	-0.23	-0.10	0.30	0.10	0.30	0.33	0.60	0.70	0.60	0.56
16	51.00	2.00	2.00	0.00	0.00	0.60	0.71	0.50	0.20	0.20	0.20	1.01	0.00	-0.03	0.07	-0.01	-0.23	-0.30	0.40	0.60	0.10	0.00	0.60	0.20	0.60	0.33
17	61.00	2.00	2.00	0.00	0.00	0.79	0.61	0.58	0.40	0.60	0.00	11.01	-0.20	0.16	-0.03	0.07	-0.03	0.10	0.30	0.10	0.00	1.00	0.30	0.20	0.10	0.00
18	56.00	2.00	2.00	2.00	2.00	0.85	0.94	1.00	0.40	0.20	0.30	6.01	0.10	0.22	0.29	0.49	-0.03	-0.30	0.20	0.30	0.90	1.00	0.80	0.50	0.10	0.00
19	52.00	2.00	1.00	1.00	0.00	0.59	0.64	0.92	0.20	0.20	0.40	2.01	0.20	-0.04	-0.01	0.40	-0.23	-0.30	0.10	0.00	0.20	0.00	0.70	1.00	0.80	0.56
20	48.00	0.00	0.00	0.00	0.00	0.71	0.79	0.17	0.20	0.60	0.30	-1.99	0.10	0.08	0.14	-0.35	-0.23	0.10	0.00	0.00	0.00	0.00	1.00	1.00	0.90	0.00
21	47.00	2.00	2.00	2.00	2.00	0.95	0.77	1.00	0.40	0.20	0.10	-2.99	-0.10	0.32	0.12	0.49	-0.03	-0.30	0.00	0.30	0.30	0.78	0.70	0.60	0.60	0.22
22	42.00	2.00	2.00	2.00	2.00	0.44	0.50	0.08	0.40	0.80	0.20	-7.99	0.00	-0.19	-0.15	-0.43	-0.03	0.30	0.30	0.00	0.00	0.11	0.50	0.60	0.90	0.89
23	42.00	2.00	2.00	1.00	0.00	0.57	0.52	1.00	0.60	0.60	0.35	-7.99	0.15	-0.06	-0.13	0.49	0.17	0.10	0.40	0.40	0.20	1.00	0.60	0.40	0.50	0.00
24	46.00	2.00	2.00	2.00	2.00	0.70	0.52	0.42	1.00	0.20	0.25	-3.99	0.05	0.07	-0.13	-0.10	0.57	-0.30	0.50	0.50	0.70	1.00	0.50	0.40	0.20	0.00
25	36.00	-999.00	-999.00	-999.00	-999.00	0.00	0.52	-999.00	-999.00	-999.00	0.05	-13.99	-0.15	-0.62	-0.13	-999.00	-999.00	-999.00	-999.00	-999.00	-999.00	-999.00	-999.00	-999.00	-999.00	-999.00
26	45.00	2.00	2.00	0.00	0.00	0.69	0.91	0.25	0.40	0.40	0.10	-4.99	-0.10	0.06	0.27	-0.26	-0.03	-0.10	0.10	0.60	1.00	1.00	0.70	0.20	0.00	0.00
27	58.00	2.00	2.00	2.00	2.00	0.44	0.54	1.00	0.80	1.00	0.20	8.01	0.00	-0.19	-0.11	0.49	0.37	0.50	0.00	1.00	1.00	1.00	1.00	0.00	0.00	0.00
28	34.00	0.00	1.00	0.00	0.00	0.60	0.29	0.08	-999.00	0.60	0.30	-15.99	0.10	-0.03	-0.36	-0.43	-999.00	0.10	0.00	0.10	0.00	0.00	0.40	0.30	0.00	0.33
29	56.00	2.00	2.00	2.00	2.00	1.00	1.00	1.00	0.40	0.40	0.00	6.01	-0.20	0.37	0.35	0.49	-0.03	-0.10	0.00	0.50	1.00	1.00	0.80	0.30	0.00	0.00
30	42.00	2.00	2.00	0.00	0.00	0.69	0.52	0.42	0.80	0.80	0.35	-7.99	0.15	0.06	-0.13	-0.10	0.37	0.30	0.00	0.50	1.00	1.00	0.80	0.30	0.00	0.00
31	58.00	1.00	1.00	0.00	0.00	0.69	0.77	0.08	0.40	0.00	0.20	8.01	0.00	0.06	0.13	-0.43	-0.03	-0.50	0.40	0.70	0.90	0.00	0.60	0.30	0.10	0.11
32	59.00	1.00	2.00	0.00	0.00	0.69	0.52	0.42	0.60	0.60	0.35	9.01	0.15	0.06	-0.13	-0.10	0.17	0.10	0.30	0.60	0.70	1.00	0.30	0.30	0.20	0.00
33	47.00	0.00	1.00	0.00	0.00	0.59	0.52	0.83	0.40	0.20	0.40	-2.99	0.20	-0.04	-0.13	0.32	-0.03	-0.30	0.30	0.90	0.80	0.78	0.60	0.10	0.20	0.22
34	51.00	2.00	2.00	2.00	2.00	0.71	0.80	0.75	0.40	0.40	0.25	1.01	0.05	0.08	0.16	0.24	-0.03	-0.10	0.70	1.00	1.00	1.00	0.30	0.00	0.00	0.00
35	42.00	0.00	0.00	0.00	0.00	0.57	0.70	1.00	0.20	0.40	0.00	-7.99	-0.20	-0.06	0.06	0.49	-0.23	-0.10	0.20	0.10	0.00	0.00	0.50	0.60	0.00	0.00
36	44.00	0.00	2.00	0.00	0.00	0.49	0.52	0.67	0.40	0.40	0.35	-5.99	0.15	-0.14	-0.13	0.15	-0.03	-0.10	0.80	0.10	0.50	0.67	0.10	0.10	0.30	0.33
101	51.00	2.00	2.00	0.00	0.00	0.60	0.64	0.00	0.40	0.20	0.25	1.01	0.05	-0.03	-0.01	-0.51	-0.03	-0.30	0.30	0.00	0.00	1.00	0.30	0.00	0.10	0.00
102	42.00	2.00	2.00	2.00	2.00	0.57	0.52	0.33	0.40	0.40	0.35	-7.99	0.15	-0.06	-0.13	-0.18	-0.03	-0.10	0.60	1.00	1.00	1.00	0.40	0.00	0.00	0.00
103	46.00	2.00	0.00	0.00	0.00	0.44	0.52	0.92	0.60	0.20	0.40	-3.99	0.20	-0.19	-0.13	0.40	0.17	-0.30	0.00	0.60	0.80	1.00	0.40	0.10	0.20	0.00
104	47.00	2.00	2.00	0.00	0.00	0.57	0.52	0.00	0.40	0.80	0.30	-2.99	0.10	-0.06	-0.13	-0.51	-0.03	0.30	0.00	0.60	1.00	1.00	0.00	0.10	0.00	0.00
105	37.00	2.00	1.00	0.00	0.00	0.44	0.52	0.83	0.60	0.40	0.25	-12.99	0.05	-0.19	-0.13	0.32	0.17	-0.10	-999.00	-999.00	-999.00	-999.00	-999.00	-999.00	-999.00	-999.00
201	54.00	2.00	2.00	2.00	2.00	0.57	0.83	1.00	0.40	0.20	0.30	4.01	0.10	-0.06	0.19	0.49	-0.03	-0.30	0.00	0.70	0.00	0.00	0.00	0.30	0.90	0.89
202	41.00	0.00	0.00	0.00	0.00	0.33	0.64	0.00	0.60	0.20	0.10	-8.99	-0.10	-0.30	-0.01	-0.51	0.17	-0.30	0.40	0.00	0.00	0.00	0.10	0.00	0.00	0.00
301	47.00	0.00	0.00	0.00	0.00	0.57	0.74	0.08	0.60	0.80	0.00	-2.99	-0.20	-0.06	0.10	-0.43	0.17	0.30	0.70	1.00	0.90	0.00	0.20	0.00	0.10	0.11
302	46.00	0.00	1.00	0.00	0.00	0.57	0.52	0.00	0.40	0.40	0.35	-3.99	0.15	-0.06	-0.13	-0.51	-0.03	-0.10	0.10	0.00	0.40	0.11	0.60	0.80	0.50	0.56
303	37.00	2.00	2.00	0.00	0.00	0.57	0.52	0.00	0.60	0.60	0.00	-12.99	-0.20	-0.06	-0.13	-0.51	0.17	0.10	0.20	0.00	0.90	0.00	0.10	0.10	0.10	0.00
304	50.00	0.00	0.00	0.00	0.00	0.57	1.00	0.00	0.80	0.80	0.15	0.01	-0.05	-0.06	0.35	-0.51	0.37	0.30	0.20	0.10	0.00	0.00	0.80	0.90	1.00	0.89
305	40.00	2.00	2.00	0.00	0.00	0.57	0.52	0.33	0.40	0.60	0.20	-9.99	0.00	-0.06	-0.13	-0.18	-0.03	0.10	0.10	0.00	0.10	0.00	0.70	0.80	0.60	1.00
306	55.00	0.00	0.00	0.00	0.00	0.56	0.71	0.42	0.40	0.60	0.40	5.01	0.20	-0.07	0.07	-0.10	-0.03	0.10	0.00	0.00	0.00	0.00	1.00	0.60	0.70	0.44
307	47.00	2.00	0.00	0.00	0.00	0.59	0.50	0.00	0.60	0.60	0.10	-2.99	-0.10	-0.04	-0.15	-0.51	0.17	0.10	0.10	0.40	0.50	1.00	0.40	0.20	0.50	0.00
309	57.00	2.00	2.00	2.00	2.00	0.79	1.00	0.17	0.40	0.60	0.05	7.01	-0.15	0.16	0.35	-0.35	-0.03	0.10	0.40	1.00	1.00	1.00	0.30	0.00	0.00	0.00
310	51.00	2.00	2.00	2.00	2.00	0.69	0.50	0.92	0.40	0.40	0.35	1.01	0.15	0.06	-0.15	0.40	-0.03	-0.10	0.00	0.00	0.00	1.00	1.00	0.80	0.80	0.00
401	57.00	2.00	2.00	2.00	2.00	0.88	1.00	0.08	0.40	0.60	0.35	7.01	0.15	0.25	0.35	-0.43	-0.03	0.10	0.10	1.00	1.00	1.00	0.80	0.00	0.00	0.00
402	44.00	2.00	2.00	1.00	2.00	0.54	0.70	0.58	0.40	0.60	0.15	-5.99	-0.05	-0.09	0.05	0.07	-0.03	0.10	0.40	0.60	0.80	1.00	0.30	0.30	0.10	0.00
403	48.00	2.00	2.00	2.00	2.00	0.91	1.00	1.00	0.40	0.40	0.40	-1.99	0.20	0.28	0.35	0.49	-0.03	-0.10	-999.00	-999.00	-999.00	-999.00	-999.00	-999.00	-999.00	-999.00
404	47.00	2.00	2.00	2.00	2.00	0.57	0.52	0.33	0.40	0.40	0.30	-2.99	0.10	-0.06	-0.13	-0.18	-0.03	-0.10	0.70	1.00	1.00	1.00	0.20	0.00	0.00	0.00
405	46.00	0.00	2.00	0.00	2.00	0.57	0.52	0.83	0.60	0.40	0.20	-3.99	0.00	-0.06	-0.13	0.32	0.17	-0.10	0.00	0.10	0.00	0.00	0.00	0.40	0.00	0.00
406	48.00	2.00	2.00	0.00	0.00	0.44	0.52	0.00	0.40	0.40	0.00	-1.99	-0.20	-0.19	-0.13	-0.51	-0.03	-0.10	0.30	1.00	1.00	1.00	0.40	0.00	0.00	0.00
407	44.00	0.00	0.00	0.00	0.00	0.79	0.50	0.17	0.00	0.60	0.00	-5.99	-0.20	0.16	-0.15	-0.35	-0.43	0.10	0.00	0.30	0.00	0.00	0.10	0.30	0.10	0.00
408	45.00	0.00	2.00	0.00	0.00	0.57	0.52	0.00	0.00	0.60	0.35	-4.99	0.15	-0.06	-0.13	-0.51	-0.43	0.10	0.20	0.00	0.00	0.22	0.80	1.00	1.00	0.78
409	38.00	2.00	0.00	0.00	0.00	0.44	0.52	0.00	0.40	0.60	0.30	-11.99	0.10	-0.19	-0.13	-0.51	-0.03	0.10	0.00	0.00	0.00	0.00	0.40	0.20	0.20	0.22
410	52.00	2.00	2.00	0.00	0.00	0.57	0.75	1.00	0.40	0.20	0.15	2.01	-0.05	-0.06	0.10	0.49	-0.03	-0.30	1.00	1.00	0.20	1.00	0.00	0.00	0.40	0.00
501	47.00	0.00	0.00	0.00	0.00	0.69	0.64	0.33	0.40	0.60	0.05	-2.99	-0.15	0.06	-0.01	-0.18	-0.03	0.10	0.50	1.00	0.90	0.44	0.40	0.00	0.10	0.11
502	47.00	0.00	0.00	0.00	0.00	0.57	0.58	0.50	0.40	0.80	0.15	-2.99	-0.05	-0.06	-0.06	-0.01	-0.03	0.30	0.50	1.00	0.20	0.00	0.10	0.00	0.10	0.00
503	53.00	2.00	2.00	1.00	1.00	0.95	0.87	1.00	0.00	0.40	0.10	3.01	-0.10	0.32	0.22	0.49	-0.43	-0.10	0.10	0.60	1.00	1.00	0.70	0.40	0.00	0.00
504	53.00	2.00	2.00	0.00	0.00	0.57	0.52	1.00	0.20	0.60	0.10	3.01	-0.10	-0.06	-0.13	0.49	-0.23	0.10	0.00	0.80	1.00	1.00	0.60	0.20	0.00	0.00
505	55.00	0.00	0.00	0.00	0.00	0.69	0.17	0.42	0.20	0.80	0.35	5.01	0.15	0.06	-0.48	-0.10	-0.23	0.30	0.90	0.80	0.90	0.78	0.10	0.20	0.10	0.11
506	53.00	0.00	2.00	0.00	0.00	0.65	0.61	0.75	0.80	0.40	0.00	3.01	-0.20	0.02	-0.03	0.24	0.37	-0.10	0.00	0.00	0.00	0.00	0.30	0.00	0.00	0.00
507	43.00	0.00	2.00	0.00	0.00	0.33	0.52	0.00	0.40	0.40	0.25	-6.99	0.05	-0.30	-0.13	-0.51	-0.03	-0.10	0.00	0.10	0.00	0.00	1.00	0.80	1.00	0.89
509	43.00	2.00	0.00	0.00	0.00	0.41	0.46	0.08	0.40	0.80	0.25	-6.99	0.05	-0.21	-0.18	-0.43	-0.03	0.30	1.00	0.60	1.00	1.00	0.00	0.20	0.00	0.00
510	39.00	2.00	0.00	0.00	0.00	0.57	0.52	0.00	0.40	0.40	0.35	-10.99	0.15	-0.06	-0.13	-0.51	-0.03	-0.10	1.00	0.30	0.00	0.11	0.00	0.10	0.10	0.56
601	57.00	0.00	0.00	0.00	0.00	0.60	0.48	1.00	0.40	0.40	0.30	7.01	0.10	-0.03	-0.16	0.49	-0.03	-0.10	0.40	0.40	1.00	1.00	0.60	0.30	0.00	0.00

602	47.00	2.00	2.00	0.00	0.00	0.82	0.84	0.58	0.40	0.40	0.25	-2.99	0.05	0.20	0.19	0.07	-0.03	-0.10	0.10	0.50	1.00	1.00	0.30	0.10	0.00	0.00
603	44.00	0.00	0.00	0.00	0.00	0.57	0.52	0.00	0.40	0.40	0.15	-5.99	-0.05	-0.06	-0.13	-0.51	-0.03	-0.10	0.00	0.30	0.20	0.00	0.70	0.60	0.40	0.00
604	43.00	2.00	0.00	1.00	0.00	0.57	0.52	0.08	0.60	0.40	0.05	-6.99	-0.15	-0.06	-0.13	-0.43	0.17	-0.10	0.30	1.00	0.90	0.00	0.20	0.00	0.10	0.00
605	58.00	2.00	2.00	2.00	2.00	0.95	1.00	1.00	0.60	0.80	0.00	8.01	-0.20	0.33	0.35	0.49	0.17	0.30	0.30	1.00	1.00	1.00	0.40	0.00	0.00	0.00
606	63.00	2.00	2.00	0.00	0.00	0.65	0.70	0.08	0.20	0.80	0.40	13.01	0.20	0.02	0.06	-0.43	-0.23	0.30	0.40	0.60	0.40	0.00	0.10	0.20	0.10	1.00
701	37.00	2.00	2.00	0.00	0.00	0.57	0.64	0.00	0.20	0.40	0.30	-12.99	0.10	-0.06	-0.01	-0.51	-0.23	-0.10	0.50	1.00	0.90	0.00	0.40	0.00	0.10	0.22
702	54.00	2.00	2.00	0.00	1.00	0.69	0.55	0.33	0.20	0.40	0.35	4.01	0.15	0.06	-0.09	-0.18	-0.23	-0.10	0.40	0.30	0.30	0.44	0.50	0.70	0.60	0.56
703	40.00	1.00	1.00	0.00	0.00	0.51	0.64	0.00	0.60	0.80	0.15	-9.99	-0.05	-0.12	-0.01	-0.51	0.17	0.30	0.00	0.00	1.00	1.00	0.60	0.60	0.00	0.00
704	50.00	0.00	2.00	0.00	0.00	0.57	0.52	0.25	0.40	0.80	0.35	0.01	0.15	-0.06	-0.13	-0.26	-0.03	0.30	0.40	0.70	0.90	0.00	0.60	0.20	0.10	0.11
705	50.00	0.00	0.00	0.00	0.00	0.37	0.52	0.42	0.20	0.20	0.20	0.01	0.00	-0.26	-0.13	-0.10	-0.23	-0.30	0.10	0.00	0.10	0.00	0.70	1.00	0.60	0.67
706	54.00	2.00	2.00	2.00	2.00	0.69	0.86	0.17	0.40	0.40	0.15	4.01	-0.05	0.06	0.21	-0.35	-0.03	-0.10	0.00	0.40	0.40	0.00	1.00	0.50	0.50	0.67
707	49.00	1.00	2.00	0.00	0.00	0.75	0.83	0.83	0.40	0.40	0.00	-0.99	-0.20	0.12	0.19	0.32	-0.03	-0.10	0.00	0.00	0.10	0.44	1.00	1.00	0.90	0.56
708	57.00	2.00	2.00	2.00	2.00	0.88	0.94	1.00	0.80	1.00	0.25	7.01	0.05	0.25	0.29	0.49	0.37	0.50	0.10	0.60	0.80	1.00	0.80	0.20	0.20	0.00
709	52.00	2.00	2.00	1.00	2.00	0.44	0.70	0.17	0.60	0.60	0.00	2.01	-0.20	-0.19	0.06	-0.35	0.17	0.10	0.60	0.00	0.00	1.00	0.10	0.00	0.10	0.00
802	46.00	2.00	0.00	1.00	0.00	0.69	0.52	0.75	0.40	0.40	0.10	-3.99	-0.10	0.06	-0.13	0.24	-0.03	-0.10	0.10	0.50	1.00	1.00	0.30	0.10	0.00	0.00
803	45.00	0.00	2.00	0.00	0.00	0.44	0.41	0.00	0.60	0.40	0.20	-4.99	0.00	-0.18	-0.24	-0.51	0.17	-0.10	0.20	0.00	0.00	0.67	0.30	0.00	0.10	0.11
804	41.00	2.00	2.00	0.00	0.00	0.66	0.61	0.33	0.80	0.60	0.15	-8.99	-0.05	0.03	-0.03	-0.18	0.37	0.10	0.30	1.00	0.40	1.00	0.50	0.00	0.40	0.00
805	46.00	0.00	0.00	0.00	0.00	0.73	1.00	0.58	0.40	0.40	0.00	-3.99	-0.20	0.10	0.35	0.07	-0.03	-0.10	0.20	0.20	0.40	0.00	0.40	0.20	0.20	0.00
806	43.00	0.00	2.00	0.00	0.00	0.69	0.52	0.17	0.40	0.80	0.40	-6.99	0.20	0.06	-0.13	-0.35	-0.03	0.30	0.10	0.10	0.10	0.00	0.90	0.70	0.80	0.67
807	37.00	1.00	0.00	0.00	0.00	0.23	0.52	0.00	0.20	1.00	0.20	-12.99	0.00	-0.40	-0.13	-0.51	-0.23	0.50	0.00	0.10	0.00	0.00	0.50	0.60	0.30	0.11
808	37.00	0.00	0.00	0.00	0.00	0.12	0.52	0.17	0.40	0.20	0.25	-12.99	0.05	-0.51	-0.13	-0.35	-0.03	-0.30	0.50	0.00	0.10	0.11	0.30	0.90	0.90	0.89
809	48.00	0.00	1.00	0.00	0.00	0.51	0.58	0.00	0.40	0.40	0.25	-1.99	0.05	-0.12	-0.06	-0.51	-0.03	-0.10	0.80	1.00	0.90	0.89	0.20	0.00	0.10	0.11
810	42.00	0.00	2.00	0.00	0.00	0.57	0.52	1.00	0.80	0.20	0.35	-7.99	0.15	-0.06	-0.13	0.49	0.37	-0.30	0.60	0.20	0.20	0.44	0.30	0.10	0.30	0.33
811	37.00	1.00	0.00	0.00	0.00	0.69	0.52	0.83	0.40	0.60	0.20	-12.99	0.00	0.06	-0.13	0.32	-0.03	0.10	0.10	0.20	0.10	0.33	0.40	0.60	0.60	0.33
812	52.00	2.00	2.00	2.00	2.00	0.61	0.66	1.00	0.20	0.60	0.35	2.01	0.15	-0.02	0.02	0.49	-0.23	0.10	0.00	0.50	0.80	1.00	0.60	0.50	0.20	0.00
813	53.00	2.00	2.00	2.00	2.00	0.67	0.52	0.25	0.40	0.40	0.10	3.01	-0.10	0.05	-0.13	-0.26	-0.03	-0.10	0.00	0.80	1.00	1.00	1.00	0.20	0.00	0.00
814	52.00	2.00	2.00	2.00	2.00	0.53	0.79	1.00	0.40	0.40	0.15	2.01	-0.05	-0.10	0.14	0.49	-0.03	-0.10	0.60	0.60	1.00	0.56	0.30	0.40	0.00	0.44
815	61.00	2.00	2.00	2.00	2.00	0.82	0.80	1.00	0.40	0.40	0.30	11.01	0.10	0.20	0.16	0.49	-0.03	-0.10	0.50	1.00	1.00	1.00	0.40	0.00	0.00	0.00
816	61.00	2.00	2.00	2.00	2.00	0.60	0.84	0.58	0.40	0.20	0.35	11.01	0.15	-0.03	0.19	0.07	-0.03	-0.30	0.40	0.90	1.00	1.00	0.40	0.10	0.00	0.00
817	55.00	2.00	2.00	1.00	1.00	0.85	1.00	1.00	0.40	0.40	0.00	5.01	-0.20	0.22	0.35	0.49	-0.03	-0.10	-999.00	-999.00	-999.00	-999.00	-999.00	-999.00	-999.00	-999.00
818	62.00	0.00	0.00	0.00	0.00	0.82	1.00	0.08	0.60	0.60	0.10	12.01	-0.10	0.19	0.35	-0.43	0.17	0.10	0.00	0.70	0.30	0.00	0.40	0.30	0.10	0.00
819	57.00	2.00	2.00	2.00	2.00	0.79	0.78	0.83	0.40	0.60	0.35	7.01	0.15	0.16	0.13	0.32	-0.03	0.10	0.20	0.60	0.50	0.56	0.60	0.20	0.40	0.22
820	61.00	2.00	2.00	2.00	2.00	0.52	0.57	0.42	0.40	0.60	0.35	11.01	0.15	-0.10	-0.07	-0.10	-0.03	0.10	0.30	0.30	0.80	1.00	0.70	0.70	0.20	0.00
821	57.00	2.00	2.00	2.00	2.00	0.65	0.84	0.92	0.40	0.40	0.05	7.01	-0.15	0.02	0.19	0.40	-0.03	-0.10	0.40	1.00	1.00	1.00	0.60	0.00	0.00	0.00
901	62.00	0.00	0.00	0.00	0.00	0.65	0.64	0.50	0.40	0.40	0.40	12.01	0.20	0.02	0.00	-0.01	-0.03	-0.10	0.10	0.00	0.00	0.11	0.80	1.00	1.00	0.78
902	61.00	0.00	2.00	0.00	2.00	0.69	0.52	-999.00	0.60	0.40	0.25	11.01	0.05	0.06	-0.13	-999.00	0.17	-0.10	0.30	0.70	0.60	0.67	0.40	0.20	0.40	0.22
903	50.00	2.00	2.00	2.00	2.00	0.75	0.68	1.00	0.40	0.60	0.25	0.01	0.05	0.12	0.03	0.49	-0.03	0.10	0.30	0.80	1.00	1.00	0.40	0.20	0.00	0.00
904	54.00	1.00	2.00	0.00	0.00	0.51	0.52	0.92	0.60	0.60	0.15	4.01	-0.05	-0.12	-0.13	0.40	0.17	0.10	-999.00	-999.00	-999.00	-999.00	-999.00	-999.00	-999.00	-999.00
906	58.00	2.00	2.00	2.00	2.00	0.79	0.81	1.00	0.60	0.40	0.10	8.01	-0.10	0.16	0.16	0.49	0.17	-0.10	0.50	0.90	1.00	1.00	0.30	0.10	0.00	0.00
907	57.00	2.00	2.00	1.00	1.00	0.44	0.52	1.00	0.20	0.60	0.25	7.01	0.05	-0.19	-0.13	0.49	-0.23	0.10	0.00	0.80	1.00	1.00	0.00	0.10	0.00	0.00
908	59.00	-999.00	-999.00	-999.00	-999.00	0.81	0.94	-999.00	-999.00	-999.00	0.30	9.01	0.10	0.18	0.29	-999.00	-999.00	-999.00	-999.00	-999.00	-999.00	-999.00	-999.00	-999.00	-999.00	-999.00
909	58.00	2.00	2.00	2.00	1.00	0.78	0.86	0.33	0.80	0.80	0.35	8.01	0.15	0.16	0.21	-0.18	0.37	0.30	0.30	0.90	1.00	1.00	0.50	0.10	0.00	0.00
910	57.00	0.00	2.00	0.00	1.00	0.95	1.00	0.08	0.40	0.60	0.05	7.01	-0.15	0.32	0.35	-0.43	-0.03	0.10	0.30	0.40	0.10	0.11	0.50	0.50	0.90	0.89
912	41.00	1.00	2.00	0.00	0.00	0.57	0.52	1.00	0.40	0.60	0.30	-8.99	0.10	-0.06	-0.13	0.49	-0.03	0.10	0.00	0.00	0.00	0.11	1.00	0.90	1.00	0.67
916	50.00	2.00	2.00	0.00	0.00	0.60	0.52	0.42	0.40	0.40	0.30	0.01	0.10	-0.03	-0.13	-0.10	-0.03	-0.10	-999.00	-999.00	-999.00	-999.00	-999.00	-999.00	-999.00	-999.00
917	38.00	1.00	1.00	0.00	0.00	0.57	0.52	0.33	0.00	0.40	0.00	-11.99	-0.20	-0.06	-0.13	-0.18	-0.43	-0.10	-999.00	-999.00	-999.00	-999.00	-999.00	-999.00	-999.00	-999.00
918	40.00	0.00	1.00	0.00	0.00	0.57	0.52	0.42	0.40	0.40	0.35	-9.99	0.15	-0.06	-0.13	-0.10	-0.03	-0.10	0.10	0.60	0.90	0.00	0.10	0.10	0.10	0.00
920	50.00	2.00	2.00	0.00	0.00	0.60	0.48	0.25	0.40	0.20	0.25	0.01	0.05	-0.03	-0.17	-0.26	-0.03	-0.30	0.10	0.10	0.00	0.56	0.90	0.40	0.50	0.44
922	45.00	-999.00	-999.00	-999.00	-999.00	0.57	0.52	-999.00	-999.00	-999.00	-999.00	-4.99	-999.00	-0.06	-0.13	-999.00	-999.00	-999.00	-999.00	-999.00	-999.00	-999.00	-999.00	-999.00	-999.00	-999.00
923	40.00	2.00	2.00	0.00	0.00	0.57	0.64	1.00	0.40	0.60	0.15	-9.99	-0.05	-0.06	-0.01	0.49	-0.03	0.10	0.60	1.00	1.00	0.67	0.40	0.00	0.00	0.22
924	48.00	2.00	2.00	0.00	0.00	0.69	0.52	1.00	0.40	0.00	0.30	-1.99	0.10	0.06	-0.13	0.49	-0.03	-0.50	0.10	0.00	0.10	1.00	0.80	0.80	0.50	0.00
925	60.00	2.00	0.00	2.00	0.00	0.78	0.88	1.00	0.40	0.40	0.05	10.01	-0.15	0.16	0.24	0.49	-0.03	-0.10	0.20	0.30	0.70	0.44	0.70	0.60	0.30	0.33
926	59.00	2.00	0.00	0.00	0.00	0.88	1.00	0.75	0.40	0.40	0.10	9.01	-0.10	0.25	0.35	0.24	-0.03	-0.10	0.80	0.80	0.60	1.00	0.10	0.20	0.20	0.00
928	55.00	0.00	2.00	0.00	0.00	0.73	0.52	0.75	0.40	0.40	0.30	5.01	0.10	0.10	-0.13	0.24	-0.03	-0.10	0.00	0.00	0.00	0.00	1.00	1.00	0.80	1.00
930	51.00	2.00	2.00	2.00	2.00	0.62	0.76	0.00	0.60	0.40	0.00	1.01	-0.20	-0.01	0.11	-0.51	0.17	-0.10	0.00	0.00	0.00	1.00	0.90	0.40	0.10	0.00
1001	55.00	2.00	2.00	2.00	2.00	0.44	0.52	1.00	0.60	0.60	0.10	5.01	-0.10	-0.19	-0.13	0.49	0.17	0.10	0.70	1.00	1.00	1.00	0.30	0.00	0.00	0.00
1002	55.00	0.00	0.00	0.00	0.00	0.51	0.50	0.17	0.40	1.00	0.35	5.01	0.15	-0.12	-0.15	-0.35	-0.03	0.50	0.00	0.00	0.00	0.22	0.00	0.00	0.10	0.11
1003	59.00	2.00	2.00	0.00	0.00	0.44	0.52	0.00	0.60	0.40	0.30	9.01	0.10	-0.19	-0.13	-0.51	0.17	-0.10	0.00	0.20	0.50	1.00	0.00	0.30	0.40	0.00
1004	43.00	1.00	1.00	0.00	0.00	0.51	0.50	0.33	0.40	0.80	0.25	-6.99	0.05	-0.12	-0.15	-0.18	-0.03	0.30	0.10	1.00	1.00	1.00	0.10	0.00	0.00	0.00
1005	41.00	2.00	2.00	2.00	2.00	0.44	0.52	0.00	0.40	0.20	0.05	-8.99	-0.15	-0.19	-0.13	-0.51	-0.03	-0.30	0.10	0.40	1.00	1.00	0.70	0.50	0.00	0.00
1006	48.00	2.00	2.00	0.00	0.00	0.69	0.50	0.83	0.00	0.60	0.30	-1.99	0.10	0.06	-0.15	0.32	-0.43	0.10	0.00	0.00	0.50	0.78	0.90	0.30	0.30	0.22
1007	49.00	2.00	0.00	2.00	0.00	0.73	0.46	0.67	0.40	0.40	0.20	-0.99	0.00	0.10	-0.18	0.15	-0.03	-0.10	0.20	0.30	0.50	0.33	0.80	0.70	0.50	0.67
1102	36.00	2.00	1.00	0.00	0.00	0.69	0.52	0.25	0.20	0.00	0.25	-13.99	0.05	0.06	-0.13	-0.26	-0.23	-0.50	0.10	0.40	0.80	0.11	0.10	0.40	0.10	0.11
1103	56.00	2.00	0.00	1.00	0.00	0.65	0.70	1.00	0.60	0.60	0.00	6.01	-0.20	0.02	0.06	0.49	0.17	0.10	-999.00	-999.00	-999.00	-999.00	-999.00	-999.00	-999.00	-999.00
1104	54.00	2.00	0.00	0.00	0.00	0.69	0.37	1.00	0.40	0.80	0.00	4.01	-0.20	0.06	-0.28	0.49	-0.03	0.30	0.20	0.10	0.50	1.00	0.10	0.20	0.10	0.00
1105	61.00	2.00	2.00	2.00	2.00	0.60	1.00	0.67	0.40	0.60	0.20	11.01	0.00	-0.03	0.35	0.15	-0.03	0.10	0.00	0.10	0.10	0.67	0.80	0.60	0.80	0.33
1106	45.00	0.00	1.00	0.00	0.00	0.57	0.52	0.75	0.60	0.60	0.35	-4.99	0.15	-0.06	-0.13	0.24	0.17	0.10	0.10	0.00	0.00	0.11	0.70	0.80	0.70	0.56
1107	53.00	2.00	2.00	1.00	1.00	0.75	0.58	0.83	0.40	0.60	0.40	3.01	0.20	0.12	-0.06	0.32	-0.03	0.10	0.30	0.30	0.20	0.89	0.10	0.50	0.10	0.11
1108	61.00	2.00	2.00	2.00	2.00	0.95	0.95	1.00	0.40	0.40	0.00	11.01	-0.20	0.32	0.30	0.49	-0.03	-0.10	0.70	0.80	1.00	1.00	0.20	0.20	0.00	0.00
1109	59.00	2.00	2.00	2.00	2.00	0.82	0.95	0.92	0.60	0.60	0.25	9.01	0.05	0.19	0.30	0.40	0.17	0.10	0.20	0.00	1.00	1.00	0.60	0.60	0.00	0.00
1110	61.00	2.00	2.00	2.00	2.00	0.57	0.52	1.00	0.40	0.20	0.30	11.01	0.10	-0.06	-0.13	0.49	-0.03	-0.30	0.00	0.00	0.00	0.11	0.90	1.00	1.00	0.89

1111	59.00	2.00	2.00	2.00	2.00	0.73	0.95	1.00	0.40	0.20	0.45	9.01	0.25	0.10	0.30	0.49	-0.03	-0.30	0.30	0.60	1.00	1.00	0.20	0.30	0.00	0.00
1112	49.00	2.00	2.00	0.00	0.00	0.57	0.52	0.58	0.60	0.80	0.10	-0.99	-0.10	-0.06	-0.13	0.07	0.17	0.30	0.20	0.00	0.20	1.00	0.10	0.00	0.10	0.00
1115	59.00	2.00	2.00	0.00	0.00	0.69	0.91	1.00	0.40	0.40	0.30	9.01	0.10	0.06	0.27	0.49	-0.03	-0.10	0.70	0.80	0.40	1.00	0.30	0.10	0.20	0.00

Missing = -999
